# Do microplastic particles affect *Daphnia magna* at the morphological, life history and molecular level?

**DOI:** 10.1371/journal.pone.0187590

**Published:** 2017-11-16

**Authors:** Hannes K. Imhof, Jakub Rusek, Michaela Thiel, Justyna Wolinska, Christian Laforsch

**Affiliations:** 1 Department of Animal Ecology I and BayCEER, University of Bayreuth, Bayreuth, Germany; 2 Department of Biology II, Ludwig-Maximilians-University Munich, Planegg-Martinsried, Germany; 3 Leibniz-Institute of Freshwater Ecology and Inland Fisheries, Department of Ecosystem Research, Müggelseedamm 301, Berlin, Germany; 4 Department of Biology, Chemistry, Pharmacy, Institute of Biology, Freie Universität Berlin, Berlin, Germany; National Taiwan Ocean University, TAIWAN

## Abstract

Microplastic particles are ubiquitous not only in marine but also in freshwater ecosystems. However, the impacts of microplastics, consisting of a large variety of synthetic polymers, on freshwater organisms remains poorly understood. We examined the effects of two polymer mixtures on the morphology, life history and on the molecular level of the waterflea *Daphnia magna* (three different clones). Microplastic particles of ~40 μm were supplied at a low concentration (1% of the food particles) leading to an average of ~30 particles in the digestive tract which reflects a high microplastic contamination but still resembles a natural situation. Neither increased mortality nor changes on the morphological (body length, width and tail spine length) or reproductive parameters were observed for adult *Daphnia*. The analyses of juvenile *Daphnia* revealed a variety of small and rather subtle responses of morphological traits (body length, width and tail spine length). For adult *Daphnia*, alterations in expression of genes related to stress responses (i.e. HSP60, HSP70 & GST) as well as of other genes involved in body function and body composition (i.e. SERCA) were observed already 48h after exposure. We anticipate that the adverse effects of microplastic might be influenced by many additional factors like size, shape, type and even age of the particles and that the rather weak effects, as detected in a laboratory, may lead to reduced fitness in a natural multi-stressor environment.

## Introduction

Microplastic particles, most often defined as particles of less than 5 mm in size, are ubiquitous in the marine environment [1–3], and contamination of the ocean with plastic debris has been characterized as one of the top emerging global issues [4, 5]. The greatest proportion of marine plastic waste originates from continental sources [6, 7] with rivers acting as major pathways [8, 9]. Lakes and streams have come more recently under focus as being similarly polluted with both macroplastic (>5 mm) and microplastic (<5 mm) particles (reviewed in [10]). For streams crossing urban and industrial zones, high loads of microplastic particles have been detected (e.g., [11, 12]). Microplastic particles can be observed in lake surface waters and beach sediments, independent of lake size, remoteness and adjacent city population or level of industrialization (e.g., [13–15]). In freshwater systems, the most commonly detected microplastic particle size class in surface waters is 5 mm down to 300 μm [16, 17], while for sediments, particles down to a few microns were identified (e.g., [11, 13, 18]). Potential introduction pathways of microplastics into lakes and streams include direct disposal, wind drift and sewage treatment plants [19, 20]. In marine ecosystems, the ingestion of microplastic particles has been demonstrated in a wide range of organisms covering different trophic levels (reviewed in [21–23]). Ingestion of microplastics by freshwater organisms is similarly likely. Evidence comes from field studies showing the presence of ingested microplastic particles and fibers in freshwater fish [24, 25], and laboratory studies proving the uptake of microplastic particles by fish and freshwater invertebrates from different feeding guilds (e.g., [13, 26, 27]).

To date several potential harms of microplastic particles were described. The release of harmful substances from microplastic particles into the digestive tract is postulated to be one of the major threats [28]. The origin of these substances can be the manufacturing process where potential toxic or endocrine disrupting additives such as Bisphenol A, are incorporated in the plastic blend. Additionally, less obvious threats may derive indirectly from plastic particles. Environmental contaminates (e.g. hydrophobic persistent organic pollutants or toxic metals) may adsorb to the surface of the particles and can be transferred to the respective organisms [29–31]. Nevertheless, the recent work of Koelmans, Bakir [32] reevaluated the potential contribution of microplastic to the transport of such substances and suggest that microplastic is of limited importance compared to other environmental media (e.g. air, soil, water). Additionally, potentially harmful microorganisms may attach to the surface or live in the biofilm covering microplastic particles [33, 34]. The biological hazard of ingested microplastic particles is enhanced due to their translocation into organs [35] and tissues [36]. This leads to a high potential for bioaccumulation, as shown in marine [37, 38] and freshwater foodwebs [39, 40]. In summary, although there is a high potential for adverse effects of microplastic particles to aquatic ecosystems, the knowledge about the impacts of microplastics, especially on freshwater organisms, is still poor [17].

The aim of this study was to establish baseline knowledge on the effects posed by environmentally relevant concentrations of microplastics on the freshwater cladoceran waterflea *Daphnia magna* at the morphological, life history and molecular level. Three clonal lines were used to further test if possible responses differ among clones. These *D*. *magna* clones were exposed to a large range of polymers commonly detected in freshwater ecosystems [10–13, 18] by supplying those in two different mixtures. Waterfleas constitute a major component of freshwater foodwebs, being not only the main food item for fish but also the main herbivore of algae, and are a well-established model organism for ecotoxicology [41]. Since only very few years, the *Daphnia* genome is available [42] and functional genomic studies are now possible. Although *D*. *magna* is primarily a filter feeder, they also graze on sedimentary algae. The latter feeding mode is used especially in the littoral zone of lakes or small ponds [43] where high accumulations of non-buoyant microplastic particles have been found [18]. Benthic feeding on microplastic particles by *D*. *magna* was already shown in laboratory experiments [13, 44]. Until now, most laboratory feeding-experiments applied high concentrations of microplastic particles (reviewed in [45]) or performed experiments with non-environmentally relevant conditions (regarding size, shape and composition of microplastics, etc. [32]). Currently published microplastic particle concentrations in freshwater environments suggest that animals will ingest microplastic particles in much lower amounts (e.g., lake surface waters: average 0.07 ± 0.08 micro- and macroplastic particles/m^2^, max: 0.22 micro- and macroplastic particles/m^2^, median: 0.04 micro- and macroplastic particles/m^2^, reviewed in [10]). In this study, *Daphnia* were exposed to a considerable low concentration of microplastic particles to food particles (1% microplastic particles in the food). This led to an ingestion rate which reflects a high microplastic contamination but might still resemble a natural situation and therefore allows for a realistic assessment of the effects of microplastics.

## Materials and methods

### *Daphnia* clones

Three different clones of *D*. *magna* were chosen from an available large collection of laboratory clones. No ethical approval is required for invertebrate use in toxicity testing. The clones originated from three different sites in Europe but all clones are established laboratory clones which have been raised in the lab during the last 6–20 years. K34J was isolated in 1998 from a former fishpond north of Munich, Germany. These fishponds were used as organic waste water treatment by a waste water treatment plant since 1929. BL2.2 originated from a small pond (Oud Meren) within a small park in Leuven, Belgium and is in culture since 1997. BL2.2 coexisted with backswimmers (*Notonecta sp*.) and fish. Max4 was hatched in 2010 from resting eggs originating from sediments from the Camargue/Tour de Valat Nature reserve, France). Predatory tadpole shrimps (*Triops cancriformis*) hatched from the same sediment. For none of the three locations data about potential plastic contamination is available. However, the highest probability for a contamination with plastic particles might exist for the fishponds near Munich which received waste water treatment plant effluent. The clones were cultured in an artificial medium based on ultrapure water, phosphate buffer, salts and trace elements [46] at 20°C ± 0.5°C and a 16h:8h light:dark regime. *Daphnia* were fed with the green algae *Scenedesmus obliquus* ad libitum.

### Tested polymers, generation of microplastic particles and stock suspensions

The three *D*. *magna* clones (BL2.2, K34J, Max4) were exposed to two different mixtures of microplastic particles, each consisting of four polymers. The tested polymers were an assortment of commonly used, non-buoyant plastic types, which are ranked among the 40 most toxic polymers [47]. All tested polymers have been detected in sediments of a subalpine lake [13, 18] and are known contaminants of freshwater ecosystems [10–12]. The polymers were assigned to two groups. Plastic mix A consisted of four polymers commonly found in freshwater ecosystems: Polyamide, polycarbonate, polyethylene terephthalate and polyvinylchloride. In contrast, plastic mix B contained polymers which, although they have a high market share, their to date detected abundance in the environment remains low: Acrylonitrile-burtdiene-styrene terpolymer, plasticized polyvinyl chloride, polyoxymethylene homopolymer and styrene-acrylonitrile copolymer [13, 18]. A detailed list of the polymers, their hazardous rank and their chemical composition are given in S1 Table. Microplastic particles were generated from raw pellets which contained a minimum number of additives necessary to produce these pellets, but without specialized additives such as pigments or plasticizers. Grinding resulted in irregular shaped particles of an average size of ~40 μm (precise size information for each polymer is provided in S1 File). The ground particles were suspended in artificial *Daphnia* medium and their concentrations were quantified as described in the S1 File.

### *Daphnia* exposure to microplastic particles

Studies assessing the abundance of microplastic plastic particles (<100 μm) in the water column using sound analytical methods are rare [10, 17]. The same is true for information about microplastic ingestion by freshwater organisms, especially zooplankton [21]. In order to expose *Daphnia* to an amount of microplastic particles resembling environmental conditions we quantified the number of algae particles during standardized feeding (S1 File) as well as performed a preliminary ingestion experiment with red fluorescent microplastic particles (S2 File). In contrast to the polymers used in plastic mix A and B, these red fluorescent particles can be easily visualized under a fluorescent microscope. The red fluorescent microplastic particles were generated from larger polymethyl methacrylate (PMMA) pellets, likewise to the particles of plastic mix A and B. Therefore, the created particles had a comparable irregular shape (S1 File) and a size of 29.5 ± 26 µm, which is similar to the size of the particles used in both plastic mixtures (~40 μm, detailed size information are available in the S1 File). The preliminary ingestion experiment revealed that a plastic particle concentration of 1% of the food particles resulted in a particle abundance of 33 ± 22 particles (mean ± SD, min: 6, max: 68) in the *Daphnia* digestive tract after 48h. This was assumed to represent a high plastic contamination but still being environmentally relevant, in contrast to previous studies using very high plastic concentrations (up to 1,000,000 particles/ml, reviewed in [45]). Nevertheless, these ingested particles numbers are higher than microplastic numbers detected in marine mussels (0.26–1.7 microplastic particles/mussel [21]), in fish from the North Sea and Baltic Sea (1–3 plastic particles/individual [48]), from the English channel (1.90 ± 0.10 particles/individual [49]), from estuarine drums (0.83 ± 0.16 particles/individual [50]). However, the assessed particle sizes in the above-mentioned studies were much larger than the particles fed in this study.

### Experimental design

**Fig 1:**
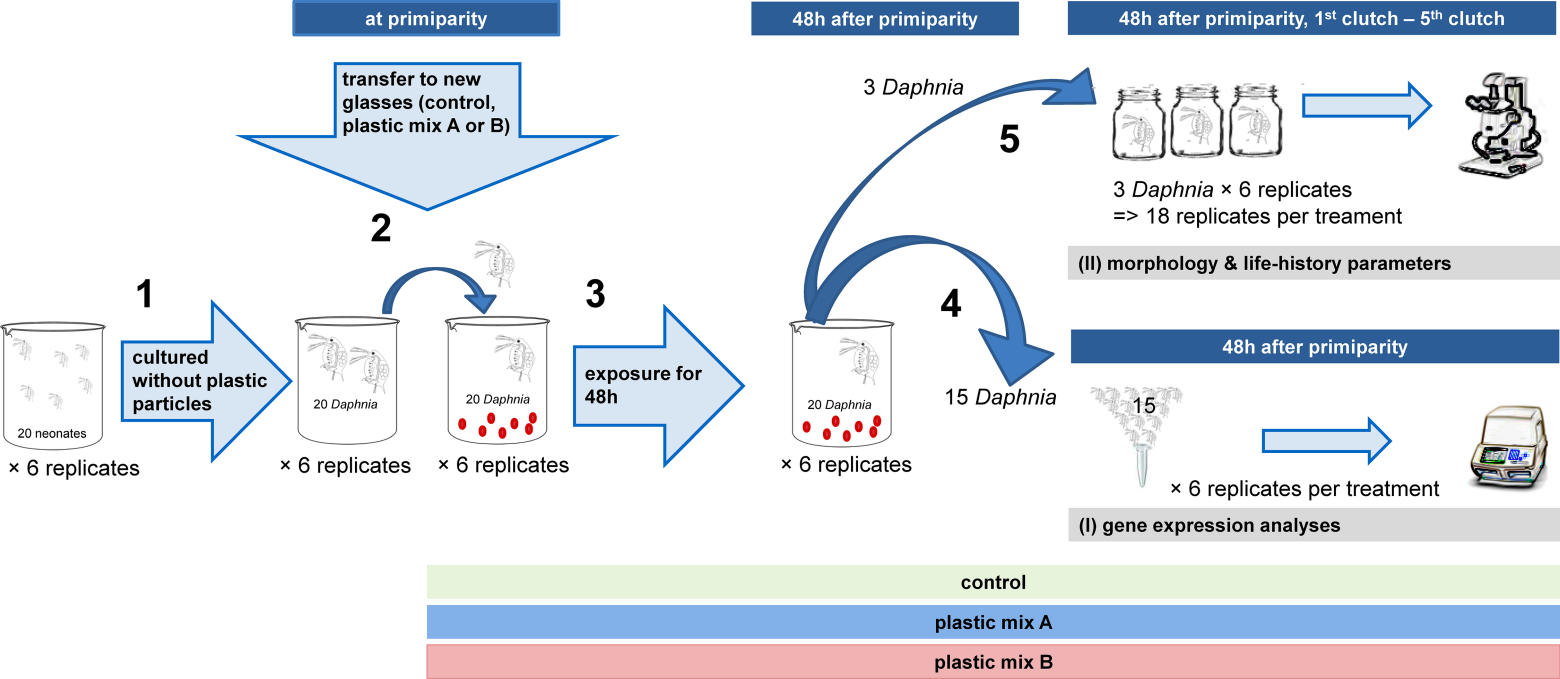
Experimental set-up for each of the three *D*. *magna* clones. 1) Initially, 20 primiparous *Daphnia* (6 replicates per clone and treatment) were cultured without any plastic particles. 2) When the animals reached primiparity, they were transferred to a glass jar prepared 24h prior with fresh artificial medium, food and the corresponding plastic mixtures. 3) They were exposed to their respective treatments for over 48 hours. 4) After the exposure, 15 of the *Daphnia* of each replicate were preserved for (I) gene expression analyses (6 replicates per treatment). 5) From the remaining five animals, three were transferred individually into small glass jars (18 replicates per treatment). These were cultured until they produced the 5^th^ brood for assessing (II) morphology and life-history parameters.

Effects of microplastic particles were assessed through two approaches (Fig 1): (I) Evaluation of gene expression after 48h exposure to microplastic particles (here, gene expression related to general stress responses, oxidative stress responses, as well as to processes such as metal detoxification, endocytosis, toxin uptake, oogenesis and juvenile development) were studied. (II) Assessment of morphological and life history parameters for adults under chronic exposure from primiparity (i.e. visible freshly deposited eggs) through the 5^th^ brood, as well as number of juveniles produced and their morphological parameters.

For evaluation of gene expression, 20 age-synchronized neonates were kept in 1.8 L of artificial medium at 20°C ± 0.5°C and a 16h:8h light:dark regime (Fig 1, step 1). To prevent microplastic contamination from the air, all experimental containers were covered with glass plates. *Daphnia* were fed with the green algae *S*. *obliquus* at a concentration of 1 mg Carbon/L every second day. A full water exchange was performed on the third day. On the sixth day, when animals were observed to have reached primiparity, plastic exposure was started. Primiparity is a well-defined and easy to observe time point which enables a standardized experiment by using age synchronized animals. The start of the plastic exposure was initiated by transferring the *Daphnia* into a jar prepared 24h prior the transfer with fresh artificial medium, food and the corresponding plastic mixtures (Fig 1, step 2), in order to allow algae and plastic particles to sink to the bottom of the experimental glasses. The sedimentation of the non-buoyant polymers and the algae ensured an equal uptake of the polymer particles by *Daphnia* from the bottom of the experimental glasses and consistent experimental conditions throughout the experiment. This feeding from the ground was already shown in a laboratory experiment [13] and in the preliminary experiment (S2 File). The plastic mixtures were supplied in a concentration which corresponds to 1% of the algae particles (290 particles/ml). This resulted in three treatments: Plastic mix A, plastic mix B and the control which did not contain any plastic particles. Each treatment of the three clones consisted of six replicates, resulting in 54 experimental units (3 clones × 3 treatments × 6 replicates; Fig 1, step 3). After 48h (i.e. 48h after primiparity, Fig 1, step 4), 15 animals from each experimental unit were preserved for gene expression analysis with Trizol (Invitrogen, USA) as described below.

For assessment of morphological and life history parameters, three of the remaining five *Daphnia* from each experimental unit were transferred individually to new 250 ml glasses. These were filled with 200 ml of artificial medium *S*. *obliquus* (2 mg Carbon/L), and stock suspensions of microplastic particles 24h prior the transfer (580 particles/ml, Fig 1, step 5). This led to 18 replicates for each treatment (plastic mix A, plastic mix B and control), which resulted in 162 experimental units (3 clones × 3 treatments × 18 replicates; Fig 1). Every other day, *Daphnia* were transferred to a new glass likewise prepared 24h prior the transfer. Animals were monitored until they produced their 5^th^ brood (Fig 1).

### Evaluation of gene expression after a 48h exposure to microplastic particles

#### RNA isolation

Fifteen animals from each replicate were transferred to a 1.5 ml Eppendorf tube, the medium was removed using a Pasteur pipette and 500 μl Trizol (Invitrogen, USA) was added. The animals were homogenized using a plastic pistil, which was thereafter rinsed with an additional 500 μl of Trizol. Samples were stored at -80°C until further processing. RNA was isolated from Trizol-preserved samples according to the manufacturer’s protocol (Isolate II RNA Mini Kit, Bioline GmbH, Germany) with the following modifications: (i) the DNase step was omitted (presence of DNA was excluded), (ii) introduction of a second drying step, (iii) elution step performed twice using half amounts of RNase-free water at the end. Extracted RNA was aliquoted in 3 tubes and stored at -80°C. Total RNA concentration and quality of the isolated RNA were assessed using a NanoDrop ND-1000 spectrophotometer (Peqlab Biotechnologie GmbH, Germany).

#### Reverse transcription of RNA and DNA contamination control

2 μg of RNA was used for reverse transcription with Tetro cDNA Synthesis Kit (Bioline GmbH, Germany) according to manufacturer instructions. In order to exclude contamination of samples with genomic DNA, a TATA-box fragment was amplified for each sample using an additional DNA positive control originating from three adult *Daphnia* (DNA isolated according to protocol described in [51]). Resulting amplicon length was checked on agarose gel, where the presence of DNA contamination could be verified based on different band length due to the presence of introns.

#### Selection of candidate and reference genes

Difference in expression level was measured based on seven candidate genes: Two genes involved in general stress response (heat shock protein, HSP 70 & 90 [52]); one gene involved in oxidative stress response (Glutathionin-S-transferase, GST [53]); two genes that are part of metal detoxification processes (Metallothionin A & B, MetA & MetB [53]); Flotilin (Flot) which has a key role in endocytosis and toxin uptake [54]; and Juvenile Hormone Esterase (JHE), which plays an important role in invertebrate oogenesis and is a key regulator of insect juvenile hormone, being responsible for controlling vitellogenesis in *Daphnia* and co-regulating the production of male offspring [55]. Increased production of male offspring is a reaction of *D*. *magna* to environmental stress [56].

Eight putative housekeeping genes were included as possible reference genes: Glyceraldehyde-3-phosphate dehydrogenase (GAPDH), succinate dehydrogenase (SDH), Syntaxin 16 (Stx16), TATA box binding protein (TBP), ubiquitin conjugating enzyme (UBC), alpha Tubulin (aTub), Actin (Act) and Sarco(endo)plasmic reticulum calcium ATPase (SERCA). SERCA is a Ca^2+^ transporter, thus playing an important role in cell calcium signaling pathways [57, 58], and is related to inducible responses in *Daphnia* to the presence of predators [59]. All these genes, with the exception of SERCA, have been previously evaluated as reference genes for *D*. *magna* qPCR assays [55, 60–62]. Reference gene stability was tested based on proven geNorm [63] and qBase technology [64]. Similar to candidate genes, genes with low stability were tested for differences in expression.

#### Primer design and validation

Primers for Act, aTub, Flot, GAPDH, GST, HSP60, JHE, MetA, MetB, SDH, UBC were obtained from [52, 53, 55, 60, 65]. For the other genes primer sequences were designed using PerlPrimer [66]. If possible, at least one primer spanned the exon/intron junction (additional control for DNA contamination). Primer pairs were tested for the presence of dimers and then optimized by end-point PCR and subsequent gel electrophoreses. A complete list of primers used in this study, the corresponding primer sequences and parameters are given in S2 Table.

#### Efficiency and specificity screening

Standard curves were produced for each of the genes to examine the efficiency of the primers and their dynamic range. For each gene, a standard curve with a fivefold dilution series from a cDNA mixture (from each clone and each treatment) was performed in three technical replicates. Standard curves were run in a Thermocycler (CFX Connect, Bio-Rad, Germany) using the following protocol: 98°C for 2 min, 98°C for 2 s, 58°C or 60°C (according to the primer temperature) for 7 s, all repeated 45 times. After every run, a melting curve analysis was performed in order to test specificity of the amplified products. The specificity of the amplicons was obtained by heating from 65°C to 95°C, in steps of 5°C. Standard curves were produced for all genes using qbase+ 2.6 (Biogazelle NV, Belgium). Single technical replicates were removed when the value varied more than one cycle from the other two. Best amplification rates were yielded at a dilution of the cDNA stock of 1:100. Primer temperature and primer concentration were optimized to R^2^ ≥0.993 and an efficiency between 92–106%, except the primers of aTub and MetA for which the efficiencies were 88.4 ± 1.5% and 87.1 ± 3%, respectively.

#### Real time–quantitative PCR analysis (RT-qPCR)

The qPCR reactions were run in technical duplicates, including a non-template control on the CFX Connect Real Time System (Bio Rad, Germany). For the qPCR reactions, 10 μl of the SensiFAST SYBR NO-Rox Kit (Bioline, Germany) was mixed with 8.4 μl of diluted cDNA (equivalent to approximately 1 μg cDNA), the corresponding amount of forward (F) and reverse (R) Primer (400 nM), and sterile water to obtain a final volume of 20 μl. The same conditions were applied in the thermal cycling program as for the efficiency screening. After every run a melting curve analysis was performed by heating from 65°C to 95°C, in steps of 5°C.

#### Statistical analyses of RT-qPCR data

All raw data were exported from the software (CFX Manager, Bio Rad, Germany) and further processed with the software qbase+ 2.6 (Biogazelle NV, Belgium). All statistical analyses were performed using qbase+ 2.6. Gene expression data were analyzed for each clone individually given clonal differences in the expression stability of reference genes. Differences of quantitation cycle (Cq) values were normalized to the reference genes and log10 transformed [67]. This in combination with the central limit theorem allows the use of parametric statistical tests and calculations [68]. Each gene was, tested using univariate ANOVA and pairwise comparisons by Tukey-Kramer for each candidate gene. The ANOVA results of each clone were corrected for multiple testing by the false discovery rate method [69]. No tests on differences between the clones were performed and therefore no further corrections for multiple testing were necessary.

### Morphological and life history parameters

The morphological parameters of the adults were recorded 48h after primiparity (prior to transfer into 250 ml glasses, Fig 1, step 5) and when *Daphnia* were carrying the 3^rd^ and 5^th^ brood. This resulted in a time of exposure of 20–22 days, depending on the duration necessary to produce the 5^th^ brood. Measurements were made using an image analyzing system (Leica MS5, Leica Mikrosysteme Vertrieb GmbH, Germany and Cell^P, Olympus GmbH, Germany). Recorded parameters were body length (distance between the upper edge of the eye and base of the tail spine), body width (longest distance between the dorsal and ventral carapace edge, perpendicular to body length) and tail spine length (distance from tip of the tail spine to its base, S1 Fig). The number of produced neonates was recorded from the 1^st^ to the 5^th^ brood. Neonates were removed from the glasses. Sex of neonates was determined under a stereo microscope for the 1^st^, 3^rd^ and 5^th^ brood. The morphological parameters of the neonates (i.e. body length, body width, tail spine length) were recorded from five randomly chosen neonates of each replicate from the 1^st^, 3^rd^ and 5^th^ brood, similar to the adults.

#### Statistical analyses of morphological and life history parameters

Statistical analyses were performed for each clone separately. Due to lacking homogeneity of variance, permutational multivariate analyses (PERMANOVA) were performed [70–72]. PERMANOVA is suitable for any multifactorial ANOVA design, allowing for all pairwise multi comparisons by permutation with nested designs and covariance analyses as well as for test designs including multiple nesting [73]. All PERMANOVA analyses were performed using Primer 6 Version 6.1.12 (Primer-E Ltd., United Kingdom) with the PERMANOVA+ package Version 1.0.2. The following settings were applied: Sums of squares type I (sequential); fixed effects sum to zero for mixed terms; permutation of residuals under a reduced model; 9999 permutations. Resemblance matrices were generated using Euclidian distances. Monte Carlo correction with 9999 permutations was used for every test. Subsequent homogeneity of dispersion was tested by separate PERMDISP tests including pair-wise comparisons in order to determine if differences between any pair of groups were due to location, spread, or a combination of the two according to the PERMANOVA user notes [71]. Resemblance matrices were generated using Euclidian distances and PERMDISP was performed with 9999 permutations on the centroid. Difference in multivariate spread were only observed for 8 out of 69 comparisons. Among the significant PERMANOVA results between the control and Treatment A or Treatment B no differences of multivariate spread were found. Detailed PERMDISD results are available in the S3 File.

The body length, body width and tail spine length of adult *Daphnia* and the neonates produced in the 1^st^, 3^rd^ and 5^th^ clutch as well as the number of produced offspring were tested for differences between the control group and animals fed with plastic mix A or plastic mix B, respectively. For the adults, PERMANOVA tests were conducted over the entire experimental period, using three different time points (48h after primiparity, carrying the 3^rd^ clutch & carrying the 5^th^ clutch) as an additional fixed factor. For the neonates as well as the number of produced offspring, PERMANOVA tests were conducted over the entire experimental period using PERMANOVA with the three different time points (1^st^, 3^rd^ & 5^th^ clutch) as an additional fixed factor and by nesting the neonates of each replicate. To compensate for size-dependent differences, analyses of body width and tail spine length of both adults and juveniles were performed using body length as a covariate. The effect of the covariate was always significant. The homogeneity of regression slopes was checked by the interaction term of the fixed factor and the covariate and was non-significant for all tests. Figures showing the body width and tail spine length of the neonates were prepared using the ratio of the body width and the tail spine length to body length in percent to compensate size-dependent differences. For figures visualizing nested data, the five individuals of each replicate were averaged to represent the nested individuals in the figures. The error bars give the 95% confidence interval which was calculated by SPSS (IBM Corp., USA).

## Results

### Evaluation of gene expression after 48h

The reference gene stability tests showed a varying gene expression among the potential reference genes between the three clones and no common reference genes could be identified due to the high interclonal differences. Therefore, normalization of gene expression was performed for each clone separately (detailed results are given in the S3 File). Three reference genes were chosen for clone BL2.2 (TBP, UBC and SDH) and Max4 (GAPDH, STX16 and UBC), and two reference genes for K34J (GAPDH and UBC).

**Fig 2:**
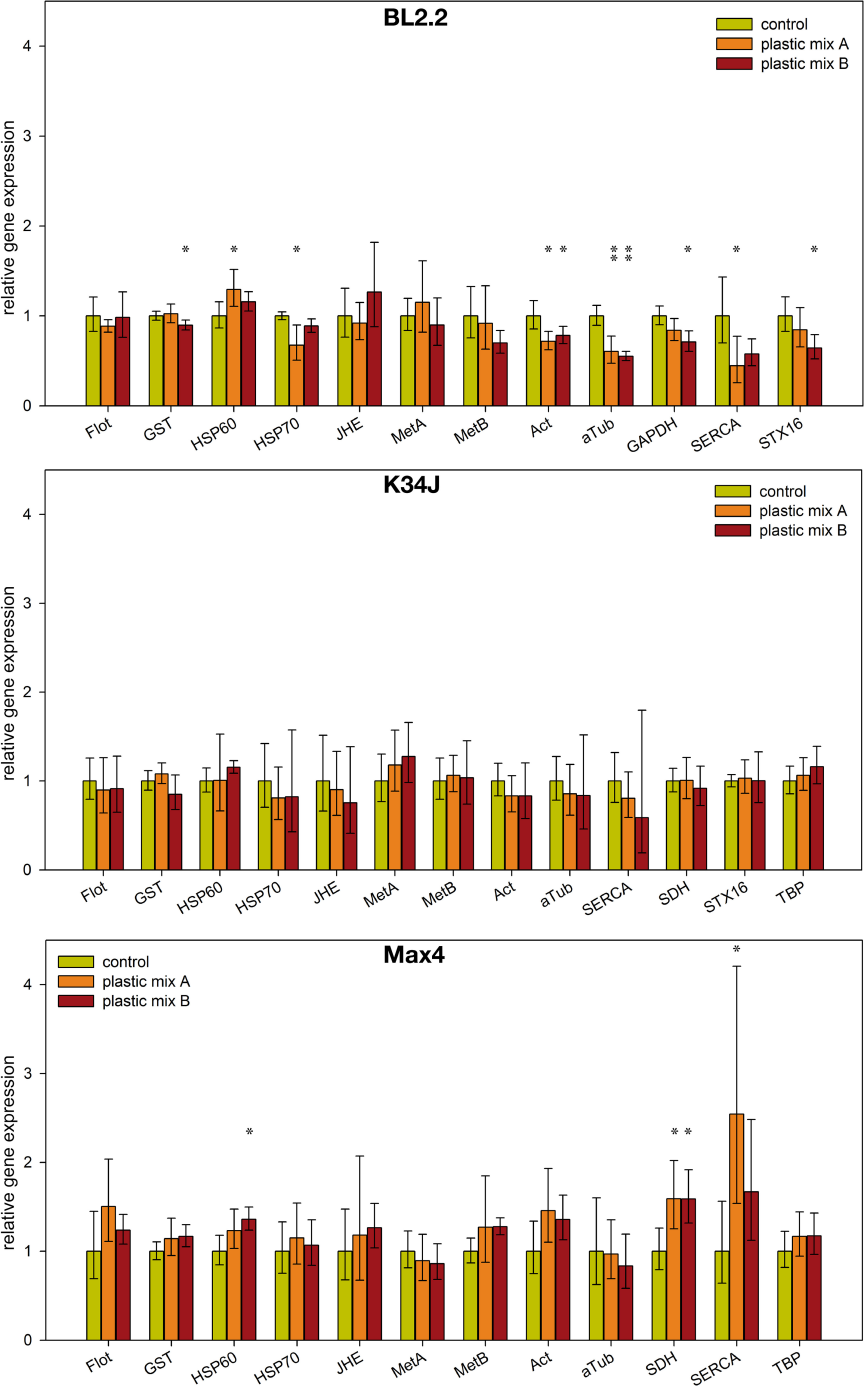
**Expression profile of *D*. *magna* clone BL2.2, K34J and Max4 after 48h exposure to plastic mix A and B.** Reference genes used for normalization of gene expression were for BL2.2: SDH, TBP and UBC; for K34J: GAPDH and UBC and for Max4: STX16, UBC and GAPDH. Error bars indicate 95% confidence interval. Confidence intervals are not affected by the correction for multiple testing by the false discovery rate method. * p<0.05, ** p<0.01.

Differences in gene expression pattern after 48h exposure to plastic mixtures were detected for clone BL2.2 and Max4, but not for K34J (Fig 2). Differentially expressed genes were involved in general stress or oxidative stress responses. However, involved were also other putative reference genes. Overall, HSP60 was up-regulated while four other genes (HSP70, Act, aTub, SERCA) were down-regulated when clone BL2.2 was exposed to plastic mix A. Exposure to plastic mix B resulted in five genes being down-regulated (GST, Act, aTub, GAPDH, STX16). The exposure of clone Max4 to plastic mix A and B led to the up-regulation of two genes in each treatment group (plastic mix A: SDH and SERCA; and plastic mix B: HSP60 and SDH). Detailed output from the statistical analysis of differential gene expression after normalization is provided in the S3 File.

### Morphological and life history parameters

**Fig 3:**
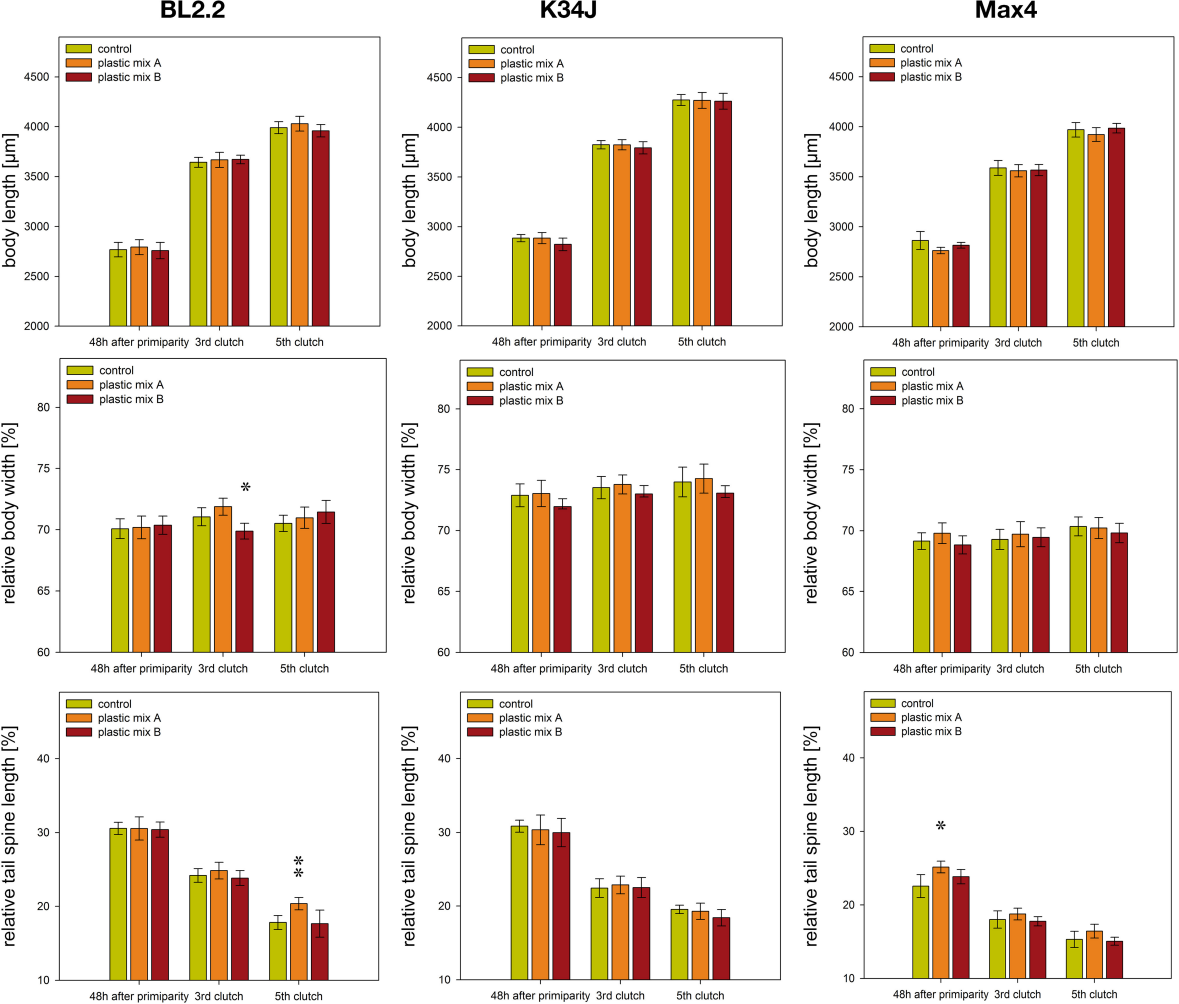
Morphological parameters of adult *D*. *magna*. Body length, body width and tail spine length of the clones Bl2.2, K34J and Max 4; measured 48h after primiparity and upon carrying the 3^rd^ and 5^th^ clutch. Statistical analyses of body width and tail spine length were performed using body length as a covariate in order to compensate for size-dependent differences. Likewise, for the figures body width and tail spine length were drawn as relative values of the body length in percent. Error bars indicate the 95% confidence intervals. Significance level against the control treatment is indicated by * p<0.05, ** p<0.01.

**Fig 4:**
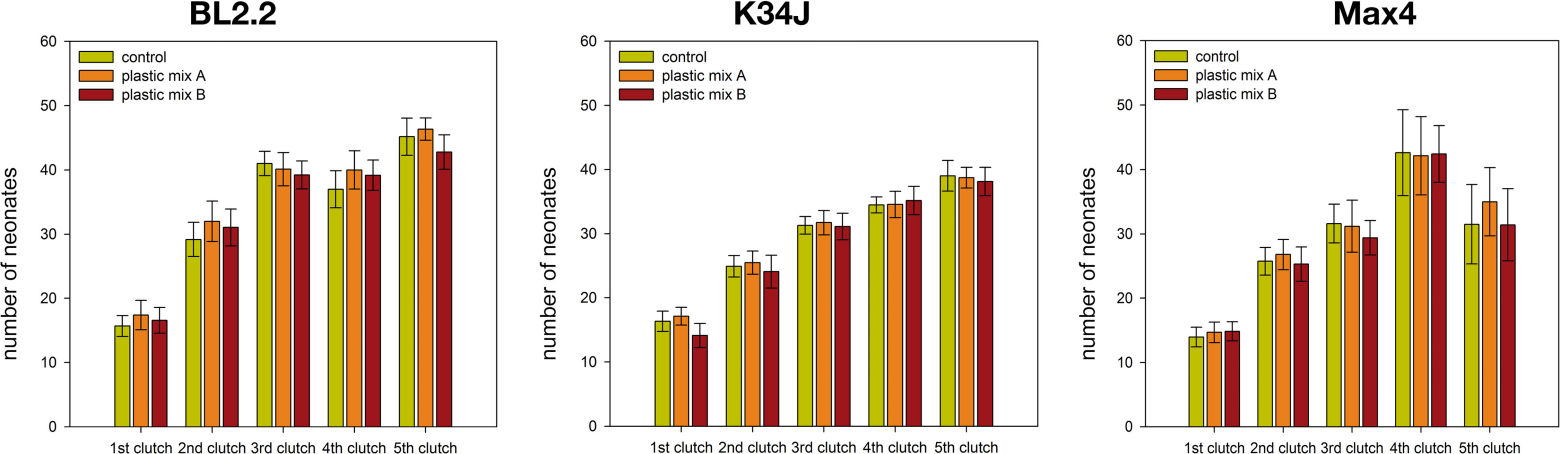
Number of offspring from *D*. *magna* produced during the entire experimental period. Error bars indicate the 95% confidence intervals.

During the entire experiment, mortality was negligible (in total, 6 out of 162 animals died, two of them were killed due to a handling error). Differences in body length were not detected between the control and plastic mix treatment groups (Fig 3, S4 File). Body width of clone BL2.2 decreased in the 3^rd^ clutch, when exposed to plastic mix B (Fig 3 & S4 File). Tail spine length was larger for two clones in plastic mix A: clone BL2.2 at the 5^th^ clutch and clone Max4 48h after primiparity (Fig 3, S4 File). Number of produced neonates did not differ between the control and plastic mix treatments (Fig 4, S4 File). Some males were produced by all three clones (0.6% of checked juveniles) but their proportion did not differ between the control and plastic mix treatments (S4 File).

**Fig 5:**
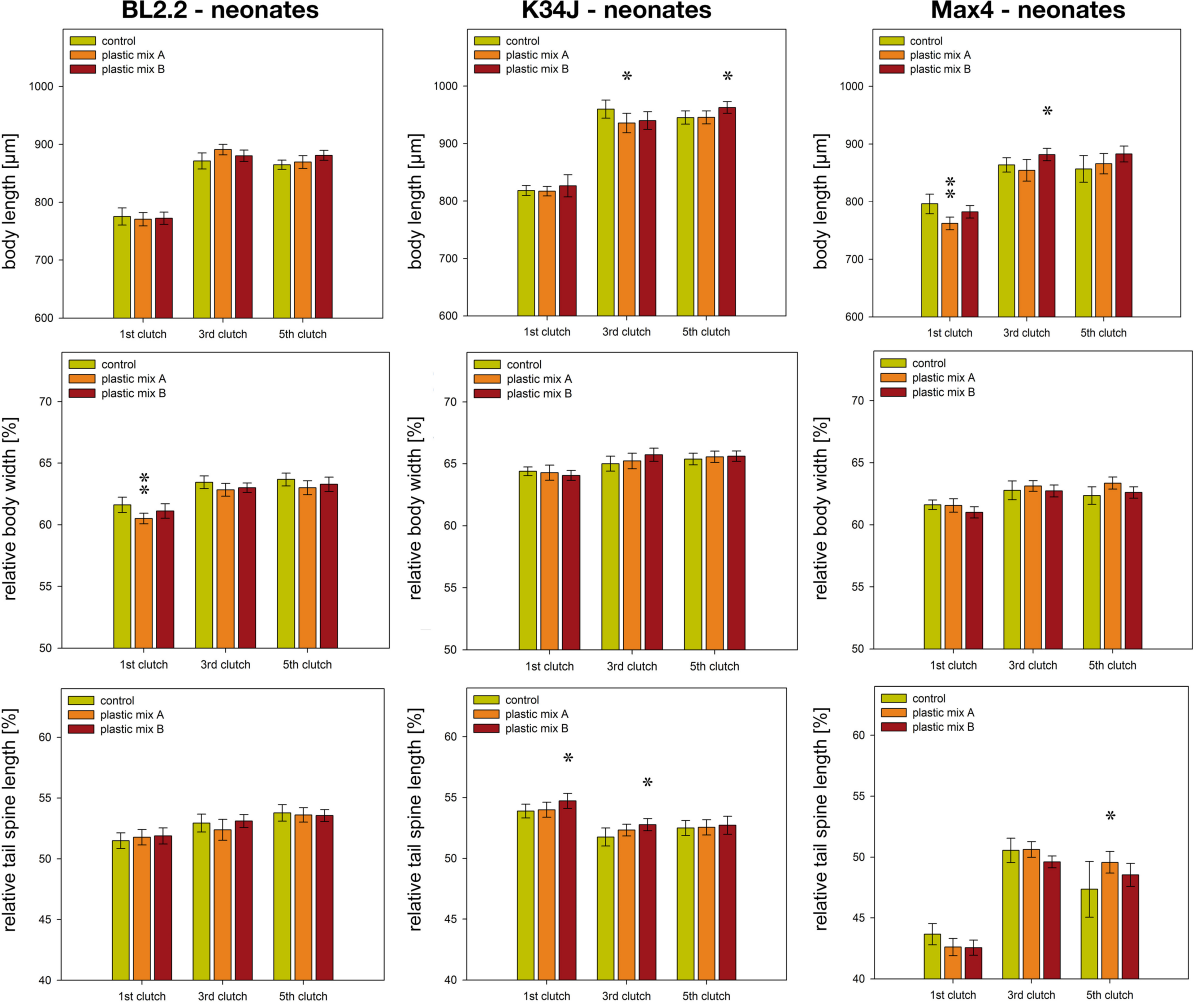
Morphological parameters of juvenile *D*. *magna*. Body length, body width and tail spine length clones Bl2.2, K34J and Max 4. The statistical analyses of body width and tail spine length were performed using body length as a covariate in order to compensate for size-dependent differences and by nesting the five individuals of each replicate. Likewise for the figures body width and tail spine length were drawn as relative values of the body length in percent. The five individuals of each replicate were averaged to represent the nested individuals in the graphs. Error bars indicate the 95% confidence intervals. Significance level against the control treatment is indicated by * p<0.05, ** p<0.01.

There were some significant, but rather inconsistent, differences in body length, body width and tail spine length within juveniles born from control and treatment mothers. For example, juvenile body length was smaller in the plastic mix A treatment for clone K34J (3^rd^ clutch) and clone Max4 (1^st^ clutch), but larger for clone K34J (5^th^ clutch) and clone Max4 (3^rd^ clutch) in the plastic mix B treatment (Fig 5, S4 File). Thinner juveniles only occurred in the 1^st^ clutch of Bl2.2 in the plastic mix A treatment group (Fig 5, S4 File). Tail spines of Max4 neonates exposed to plastic mix A in the 5^th^ clutch were longer than the tail spines of the control group (Fig 5, S4 File). Similarly, neonates of K34J exposed to plastic mix B had longer tail spines than the control group in both the 1^st^ and 5^th^ clutch (Fig 5, S4 File).

## Discussion

Ingestion of microplastic particles from both plastic mixtures, at an adopted concentration resembling a realistic ingestion rate, did not increase mortality of *Daphnia*. This is in concordance with described reaction of *D*. *magna* to 1-μm polyethylene beads, but these 1-μm particles caused increasing immobility with time and dose (EC50 57.43 mg/L after 96h [44]). However, immobility was not observed in our study. Likewise, experiments on marine species evoked little or no mortality, when organisms were exposed to plastic polymers consisting of a single substance (reviewed in [23, 74]). For example, no acute toxicity was observed in the marine isopod *Idotea emarginata* or the marine copepod *Tigriopus japonicus* when exposed to polystyrene microparticles [75, 76]. Additionally, almost no changes in body size of the adult *D*. *magna* were observed here. Likewise, growth and intermolt duration of the marine isopod *I*. *emarginata* did not change after exposure to microplastic particles and fibers [75]. Contrary to the copepod *T*. *japonicus*, which showed a significant decrease in fecundity [76], the number of produced juveniles did not change for *D*. *magna*. Decreased fecundity in *T*. *japonicus* might have resulted from the higher concentrations used in the exposure experiments compared to the adopted concentrations in this study. Nevertheless, it cannot be ruled out that other parameters such as lipid content of the eggs might be affected by microplastic exposure.

Although neither morphology nor life history of *D*. *magna* adults were affected by microplastics, offspring showed a variety of significant but small differences in body length, body width and tail spine length. Strong responses, such as increased mortality or malformations after exposure of *D*. *magna* to nano-polystyrene [40], were not observed. Depending on the clone, the juveniles in some clutches were smaller and in others larger compared to the control group. A comparison of both, life-history and morphological traits of adult and juvenile *D*. *magna* among the clones showed inconsistent results. However, this cannot be regarded as influence of the microplastic particles, as the reaction of the control treatments of the corresponding clones showed a comparable high variation. The above mentioned observations correspond to frequently described results from *Daphnia* ecotoxicity tests of pharmaceuticals with low acute toxicity, where only subtle effects occur after chronic exposure or the effects are only observed in future generations [77, 78].

Changes in offspring body size but also the elongation of tail spines are a common phenotypic plastic defenses of *Daphnia*. [46, 79, 80]. Both were observed in a present study, at different time points and in different clones after exposure to both plastic mixes whereas such defences should normally only be expressed if predators are present. Displaying the wrong defensive strategy against the actual predator spectrum might lead to predator induced mortality [81]. Hence, even if changes in body size as well as alteration in tail spine length could be regarded as subtle effects, they may have an impact on *Daphnia* survival if considered in an ecological context.

The morphological data of both adult and juvenile *Daphnia* suggest that only some of the assessed parameters were affected and that occurring effects are small and subtle. The gene expression data indicate an increased stress level of adult *Daphnia* of some clones after only 48h exposure to both plastic mixtures, although also here the observed changes were rather small. In addition, similar to the morphological data, the interclonal variation in gene expression between the three clones was high, as likewise described in other *Daphnia* studies evaluating differences in gene or protein expression [82–84]. The strongest reactions to microplastic particle exposure were observed for the clone BL2.2. However, high variability in gene expression between replicates may have obscured weak effects in the other clones. Common stress genes from the HSP family were differentially expressed under microplastic exposure, compared to the control treatment, in the clones BL2.2 and Max4. Both HSP60 and HSP70 are part of the intracellular alarm and repair system which protects protein integrity against negative effects induced by environmental stressors like heat or toxicants [84]. The upregulation of HSP60 has been documented in *D*. *magna* after 24h exposure to NO_3_ coated silver nanoparticles at a concentration of 1/4 LC_25_ [53]. Some studies observed a return of HSP60 to base levels within 24 hours, probably due to the energetic costs of maintaining high HSP levels over long periods [82, 85]. In combination with the fact that maximum expression levels can be reached after only 6h of exposure [82, 85], this could indicate that assessment of expression for this particular gene after 48 hours was too late and some individuals may have already returned to base levels. This could have led to the high variability in HSP60 levels observed between replicates in the present study. In contrast to HSP60, HSP70 is regarded as a chronic stress marker which maintains high levels even after long-term exposure [86]. Despite this, the only detected response of HSP70 was a downregulation in clone BL2.2, in the plastic mix A treatment. A similar downregulation of HSP70 was observed after exposing *T*. *japonicus* to environmental toxicants (4-nonylphenol and 4-t-octylphenol), indicating that the downregulation of HSP70 can be utilized as a sign of stress [86]. Nevertheless, the limited HSP70 reaction to both plastic mixtures, as observed in our experiment, could be due to the recently suggested ability of *Daphnia* to rapid microevolution, leading to the acquisition of toxicant resistance. This was suggested in another study [84], which found a correlation between cadmium sensitivity in different clones of *D*. *magna*, cadmium accumulation and expression of HSP70 in these clones. Varying susceptibility to plastic exposure within the three clones could be due to increasing persistence of microplastic particles in the environment over the last few decades and resulting adaptation to related compounds. However, no record of microplastic particles exists for the field sites where clones were isolated from. Only for the origin of K34J, a fishpond used for organic treatment of waste water by a nearby waste water treatment plant, a contact with microplastic particles can be anticipated. Nevertheless, genes from the HSP family could remain a proxy for stress responses in *D*. *magna*. In addition to general stress genes, the gene GST was downregulated in BL2.2. As GST is an enzyme which removes reactive oxygen species from cells [87], this indicates that microplastic particles may also interact with pathways related to oxidative stress responses. Exposure of mussels (*Mytilus galloprovincialis*) to virgin as well as pyrene treated polyethylene and polystyrene microparticles revealed an inhibition of anti-oxidant responses [88]. Though, the GST family is quite large and not all GST genes show the same strong reaction to oxidative reagents like H_2_O_2_ or trace metals (e.g., [89]), it remains worthwhile to include genes from the GST family in gene expression bioassays. Interestingly, MetA and MetB genes showed no differential expression in any of the studied clones, despite the fact that both genes are known to respond to metal exposure [90]. Metals might be a compound of plastic blends or they can be transported by plastic particles as an adsorbed environmental contaminant [18, 30]. The downregulation of the gene SERCA for the clone BL2.2 and the upregulation in Max4 after exposure to plastic mix A is intriguing. SERCA was initially intended as a reference gene, given that it plays a role in cell calcium signaling during *Daphnia* response in the vicinity of a predator presence [57, 59]. Therefore, SERCA was expected to be equally expressed across all treatments as no predators were present during the experiments. The observed upregulation of SERCA in clone Max4, in combination with elongated tail spines in adult *Daphnia*, might indicate an interference in the signaling pathway responsible for inducing anti-predation responses. SERCA is known to influence cuticle composition during expression of inducible defenses like carapace fortification [57, 59, 91]. This is further supported by observed changes in juvenile body size and elongation of tail spines, both common anti-predation responses [46, 79, 80]. A similar interaction of nano-polystyrene chemical cues inducing phenotypic plastic defenses in *D*. *magna* has already been suggested [40]. Nevertheless, it is not clear if microplastic particles interact with anti-predation responses and what the mechanisms are. They might act directly on morphological traits or leaching additives might interact with the signaling pathway responsible for inducing phenotypic plastic responses in defensive traits. Such an influence of polystyrene microplastic particles on anti-predation responses was shown in fish larvae; small behavioral changes resulted in an altered predator avoidance and a reduced survival rate [92]. However, the mechanism behind this interference remained unclear.

### Conclusion

To the best of our knowledge, this is to date the first study exposing *Daphnia* to irregular shaped microplastic particles which contained a minimum number of additives necessary to produce them, but without specialized additives (e.g. pigments, plasticizers) or environmental pollutants. In contrast to many other studies microplastic particles were supplied in amounts resulting in low ingestion rates. While low, these rates are more realistic considering currently published contamination levels in freshwater systems.

None of the three *D*. *magna* clones tested here were severely affected by microplastic exposure; neither an increased mortality nor malformations of the adults or the juveniles were detected. Nevertheless, several small and subtle but also inconsistent effects were detected on both the morphological and the molecular level. It is possible that these subtle effects might be due to natural variation or random effects and therefore *D*. *magna* species does not respond to the used microplastic particles. Nevertheless, we cannot exclude that environmental concentrations of microplastics will pose a threat to *Daphnia*, as the toxicity might be influenced by many additional factors like size, shape, type and even age of the particles.

Our study sets a baseline for future studies of microplastic particles examining more complex scenarios; concerning the shape, size, additive content or adsorbed chemical compounds in an environmental multi-stress context, with natural stress on one hand (predation pressure, interspecific and intraspecific competition, etc.) and the joint effect of anthropogenic stress (pollutants, rising CO_2_ levels and temperature, etc.) on the other hand. To conclude, further research (e.g. long-term experiments, multi-generation studies, multi-stressor experiments) are necessary to assess the effects of microplastic particles, as superficially weak effects, as observed in this laboratory environment, could have an impact at both the individual or population level in nature.

## Supporting information

S1 Fig Measurement of the body morphology of a primiparous *D*. *magna* (clone Bl2.2). (PDF)

S1 Table Polymer affiliation to plastic mix A or B, hazardous rank and most toxic chemicals. (PDF)

S2 Table RT-qPCR parameters of candidate reference genes and target genes investigated. (PDF)

S1 File Microplastic particles: Generation, stock suspensions, particle quantification and characterization. (PDF)

S2 File Preliminary experiment: Ingestion of microplastic particles and establishment of experimental concentration. (PDF)

S3 File Supporting results: Evaluation of the gene expression after 48h of exposure to microplastic particles. (PDF)

S4 File Supporting results: Morphology and life-history parameters. (PDF)
