# Supplementary material for: Do microplastic particles affect Daphnia magna at the morphological, life history and molecular level?
Source: PLoS One. 2017 Nov 16;12(11):e0187590. doi: 10.1371/journal.pone.0187590 (PMC5690657; doi:10.1371/journal.pone.0187590)
Supplement: S1 Fig — (PDF) [file pone.0187590.s001.pdf]

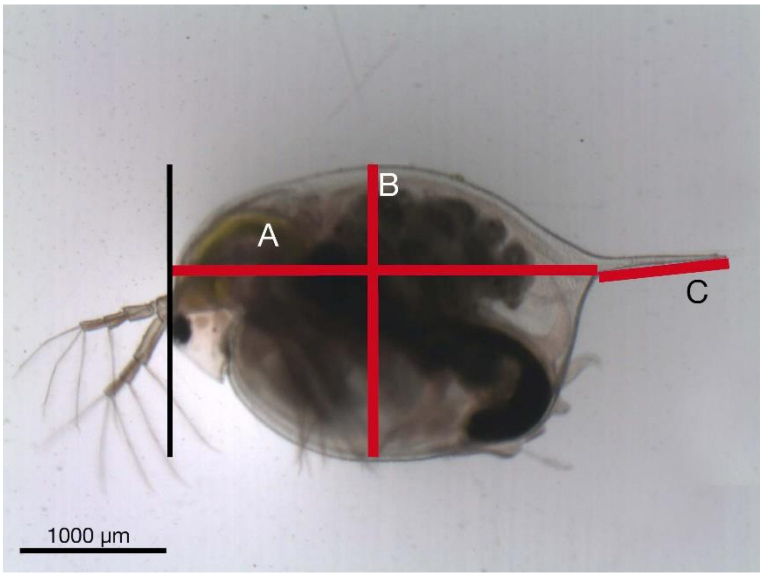

S1 Fig. Measurement of the body morphology of a primiparous *D. magna* (clone Bl2.2).
A) Body length, B) body width and C) tail spine length. The black line is an auxiliary line marking the upper corner of the eye.
