# Supplementary material for: Do microplastic particles affect Daphnia magna at the morphological, life history and molecular level?
Source: PLoS One. 2017 Nov 16;12(11):e0187590. doi: 10.1371/journal.pone.0187590 (PMC5690657; doi:10.1371/journal.pone.0187590)
Supplement: S1 Table — (PDF) [file pone.0187590.s002.pdf]

S1 Table. Polymer affiliation to plastic mix A or B, hazardous rank and most toxic chemical. Information are given according to Lithner et al. (2011). Excluding endocrine disruptors as these are until now not listed in the globally harmonized system of classification and labelling of chemicals.

Plastic mix	Polymer	Rank	Monomers	Effect
A	polyamide family	33 and lower	various e.g.<br>Adipic acid (56wt%)<br>Hexamethylenediamine (44 wt%)<br>Sebacic acid (63 wt%)<br>11-Aminoundecanoic acid (100 wt%)<br>Lauryl lactam (100 wt%)	 Eye irrit. 2 (II)<br>Skin Corr 1B (III), Acute Tox, 4[o,d] (II) STOT SE 3[ri] (II)<br>not classified<br>not classified<br>not classified
A	polycarbonate with phosgene or diphenyl carbonate*	19 or 28	BisphenolA up to 70 wt%,<br>Phosgene up to 30 wt%<br>Diphenyl carbonate up to 50 wt%	**Skin Sens. 1 (IV)**, Repr. 2[f] (III), Eye Dam. 1 (III), STOT SE 3[ri] (II) under discussion endocrine disruptors<br>**Acute Tox. 2[i] (IV),** Skin Corr. 1B (III)<br>not classified
A	polyethylene terephthalate (PET)	36	the monomers used to produce PET were not classified so there is some uncertainty, however, the SIDS Initial assessment reports indicated low level of concern<br>Ethylene glycol (39 wt%)<br>Terephtalic acid (63 wt%)<br>Dimethyl terephthalate (61 wt%)	 Acute Tox. 4[o] (II)<br>not classified<br>not classified
A	polyvinyl chloride rigid	7	Vinyl chloride (100wt%)	**Carc. 1A (V),** Flam Gas 1 (I)
B	acrylonitrile-butadiene-styrene (ABS) terpolymer	10	Styrene (58 wt%),<br>Acrylonitrile (22 wt%)<br>1,3 Butadiene	under reevaluation: acute Tox 4[oi] (II) Eye Irritation 2 (II)<br>**Carc. 1B (V), Skin Sens. 1 (IV)**, Acute Tox. 3[o,d,i] (III), Eye Dam. 1 (III), Aq. Chronic 2 (III), STOT SE[ri] (II), Skin Irrit. 2 (II), Flam Liq. (I)<br>**Carc. 1A (V), Muta. 1B (V**), Flam. Gas I (I)
B	plasticized polyvinyl chloride *	5 or 11	Vinyl chloride (50 wt%)<br>Most toxic plasticizer: Benzyl butyl phthalate (BBP (50 wt%)<br>plasticizer unknown up to 50 wt% (DIDP, DINP, etc.)	**Carc. 1A (V)**, Flam Gas 1 (I)<br>**Repr. 1B[FD], Aq. Chronic 1 (IV)**, Aq. Acute 1 (III)<br>under reevaluation: endocrine disruptors
B	polyoxymethylene (POM) homopolymer	16	Formaldehyde	**Skin Sens. 1 (IV)**, Carc. 2 (III), Acute toxicity 3[o,d,i] (III), Skin Corr. 1B (III)
B	styrene-acrylonitrile (SAN) copolymer	14	Styrene (76 wt%)<br>Acrylonitrile (22 wt%)	under reevaluation: Acute Tox. 4[f] (II), Eye Irrit 2 (II), Skin Irrit. 2 (II)<br>**Carc. 1B (V), Skin Sens. 1 (IV)**, Acute Tox. 3[o,d,i] (III), Eye Dam. 1 (III), Aq. Chronic 2 (III), STOT SE[ri] (II), Skin Irrit. 2 (II), Flam Liq. (I)

*could be ranked as more hazardous if endocrine disruption is identified
[o,d,i]Toxic by **o**ral, **d**ermal and **i**nhalation route, applies to Acute Tox. categories
[r,i] may cause respiratory irritation, applies to STOT SE 3 classification
[f]may damage fertility and the unborn child, applies to Repr. Classes
wt.% percentage by weight
Level V and level IV classification are in bold

Reference: Lithner, D., Larsson, Å., Dave, G. 2011. Environmental and health hazard ranking and assessment of plastic polymers based on chemical composition. Science of The Total Environment 409: 3309-3324.
