# Supplementary material for: Do microplastic particles affect Daphnia magna at the morphological, life history and molecular level?
Source: PLoS One. 2017 Nov 16;12(11):e0187590. doi: 10.1371/journal.pone.0187590 (PMC5690657; doi:10.1371/journal.pone.0187590)
Supplement: S2 Table — (PDF) [file pone.0187590.s003.pdf]

S2 Table. RT-qPCR parameters of candidate reference genes and target genes investigated. For Amplicon size calculation, the primers were located on the transcriptome and the distance between them was measured.

Gene name	Symbol	Gene ID	wfleaBase	Temperature. [°C]	[a,b]Efficiency	[b]R^2	Forward primer	Reverse primer	Amplicon size [bp]	Source
Actin	Act	AJ292554		58	1.968 ± 0.010	0.999	CCACACTGTCCCCATTTATGAA	CGCGACCAGCCAAATCC	71	Heckmann et al. (2006)
Alpha- tubulin	aTub			60	1.884 ± 0.015	0.998	TGGAGGTGGTGACGACT	CCAAGTCGACAAAGACAGCA	89	Heckmann et al. (2006)
Flotilin protein	Flot	DM00331		60	1.997 ± 0.03	0.996	GCGACCAATTCGCTTCTCTA	TGGTGAAGGAAAGGATTTCG	81[#]	Poynton et al. (2012)
Glyceraldehyde-3-phosphate dehydrogenase	GAPDH	AJ292555		58	1.946 ± 0.006	1.000	GGCAAGCTAGTTGTCAATGG	TATTCAGCTCCAGCAGTTCC	89	Heckmann et al. (2008)
Glutathionin-S-transferase S1	GST	DV437830		58	1.932 ± 0.013	0.999	TCAGGCTGGTGTTGAGTTTG	GAGCAAGCATTTGTCCATCA	119[#]	Poynton et al. (2012)
Heat shock protein 60	HSP60			60	1.993 ± 0.008	1.000	AGCTGGTGATGGAACAACTG	TGAGATAGTGGCAACCTGAG	-*	Steinberg et al. (2010)
Heat shock protein 70	HSP70		Dapma7bEVm002643t1	56	1.969 ± 0.032	0.995	TGTCCAGACTTACCATAAGCA	CAACGTCAAGCAACAAAGGA	103	this study
Juvenile hormone esterase	JHE	BJ932560		60	1.932 ± 0.012	0.999	ATGGAGTTCTCAACGGAACG	ATCTCAGGTGTGGGCATTTC	100	Heckmann et al. (2008)
Metallothionin A	MetA	DV437799		60	1.871 ± 0.030	0.993	TTGCCAAAACAATTGCTCAT	CACCTCCAGTGGCACAAAT	134#	Poynton et al. (2008)
Metallothionin B	MetB	DV437826		60	1.924 ± 0.031	0.994	GTGGAACCGAATGCAAATG	TGCATGGACAACTGGAACTG	123#	Poynton et al. (2008)
Succinate dehydrogenase	SDH		Dapma7bEVm027685t1	60	2.017 ± 0.019	0.998	TGCCATTTAGTCGCACTCAG	GTGAGCTTGTCCTCCTTTGC	89	Heckmann et al. (2006)
Sarcoplasmic Calcium ATPase	SERCA		Dapma7bEVm010346t1	60	1.932 ± 0.012	0.999	TCGCAAAGAAGTCTTCGACTC	AAACACCAATACGACGGCAG	125	this study
Syntaxin 16	STX16		Dapma7bEVm006557t1	60	1.942 ± 0.018	0.998	TCTATCCTATCCAGGACAGTGC	AAACTCGTCTTTCGTTGAACAG	131	this study
TATA-box binding protein	TBP		Dapma7bEVm004355t1	60	2.067 ± 0.011	1.00	TGTGAGTCAGAACGAGACCT	AAGAAGATTCAAGATTAGCAGCCC	120	this study
Ubiquitin Conjugating Enzyme	UBC	BJ928996		60	1.998 ± 0.016	0.999	TCACCTGCACTCACCATTTC	AATCTCCGGAACCAAAGGAT	90	Heckmann et al. (2006)

[a]Values are mean ± SD, [b]as determined/calculated in this study,
-* not available
[#]Values were not given in the cited publication and calculated like for the self-designed primers.

References:

Heckmann, L.-H., Connon, R., Hutchinson, T. H., Maund, S. J., Sibly, R. M., Callaghan, A. 2006. Expression of target and reference genes in *Daphnia magna* exposed to ibuprofen. BMC Genomics 7: 175.

Heckmann, L.-H. et al. 2008. Systems biology meets stress ecology: Linking molecular and organismal stress responses in *Daphnia magna*. Genome Biology 9: R40.

Poynton, H. C., Loguinov, A. V., Varshavsky, J. R., Chan, S., Perkins, E. J., Vulpe, C. D. 2008. Gene expression profiling in *Daphnia magna* Part I: Concentration-dependent profiles provide support for the no observed transcriptional effect level. Environmental Science & Technology 42: 6250-6256.

Poynton, H. C. et al. 2012. Toxicogenomic responses of nanotoxicity in *Daphnia magna* exposed to silver nitrate and coated silver nanoparticles. Environmental Science & Technology 46: 6288-6296.

Steinberg, C. W., Ouerghemmi, N., Herrmann, S., Bouchnak, R., Timofeyev, M., Menzel, R. 2010. Stress by poor food quality and exposure to humic substances: *Daphnia magna* responds with oxidative stress, lifespan extension, but reduced offspring numbers. Hydrobiologia 652: 223-236.
