# Supplementary material for: Do microplastic particles affect Daphnia magna at the morphological, life history and molecular level?
Source: PLoS One. 2017 Nov 16;12(11):e0187590. doi: 10.1371/journal.pone.0187590 (PMC5690657; doi:10.1371/journal.pone.0187590)
Supplement: S1 File — (PDF) [file pone.0187590.s004.pdf]

S1 File. Microplastic particles: Generation, stock suspensions, particle quantification and characterization

Generation of microplastic particles

Microplastic particles for feeding experiments were produced from plastic pellets of polyvinyl chloride (soft & rigid), polystyrene, polyamide, polycarbonate, acrylnitril-butadien-styrol-copolymerisat, polyoxymethylen, styrol-acrylnitril and red fluorescent polymethylmethacrylat. For polyethylene terephthalat it was not possible to buy pre-production pellets. Therefore, several plastic bottles were cut into small pieces and were grinded according to the same protocol.

Plastic pellets of the above mentioned polymer types were frozen to -60°C in Falcon Tubes (VWR International GmbH, Germany). Subsequently, these were grinded with the use of a commercial kitchen grinder (Professional Mixer 600 W, Elektrogeräte Solac Vertrieb GmbH, Germany) with the addition of aqua bidest and ice in order to keep the suspension at low temperature. The mixture was then filtered over a gauze with a pore size of 100 µm, transferred with a spatula to a glass petri dish (diameter 30 cm) and dried at 40°C. The dried powder was further separated with analytical stainless steel sieves (DIN 4188, Retsch GmbH, Germany) into particles >630 µm, 630 – 200 µm and <200 µm. Particles from the finest fraction were further grinded using a ball mill (Mikro-Dismembranator S, B. Braun, Biotech International, Germany or MM400, Retsch GmbH, Germany). Grinding success was dependent on the physical properties of the polymers but resulted in irregular shaped fragments of all polymers.

Characterization of microplastic particles used in the experiments

Particle shape

All microplastic particles of plastic mix A, plastic mix B and the red florescent particles made of polymethyl methacrylate (PMMA) were generated from raw pellets of the respective polymers in a similar way. Therefore, grinding resulted in comparable irregular shaped fragments with a comparable size (Fig. S1, S3 & S4, Table S1).

Particle size was measured on a microscopy slide cleaned with Ethanol (70%) using a digital image analyzing system (Leica MS5 in combination with Cell^D, Olympus European GmbH, Germany). All particles in the field of view, using a magnification of 2.5x (590.92 mm²) were imaged and measured. The measurements of all particles were averaged and the standard deviations calculated. The detailed measurements are given in Table S1

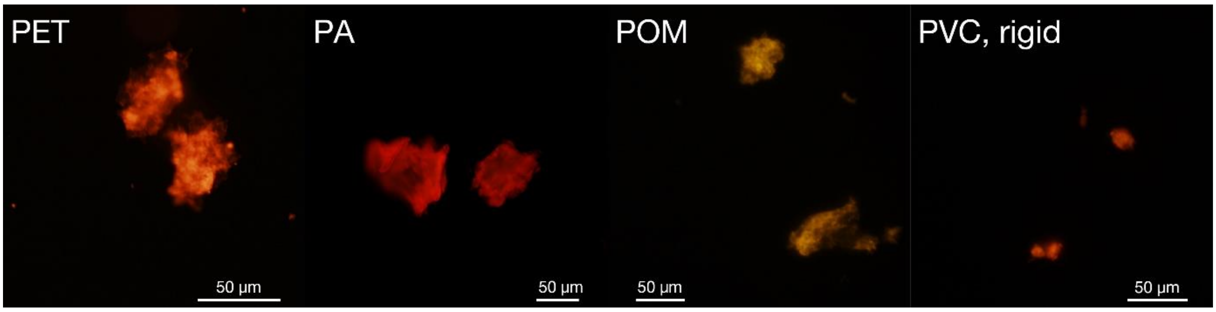

Fig. S1. Fluorescence microscopy images of microplastic particles from plastic mix A: Polyethylene terephtalate (PET), polyamide (PA), polyoxymethylene homopolymer (POM) and rigid polyvinylchloride (PVC, rigid). The particles were not stained with fluorescence dye, but images show their auto-fluorescence in order to increase the contrast. Particles were imaged under an Olympus BX61 with GFP-LP filter.

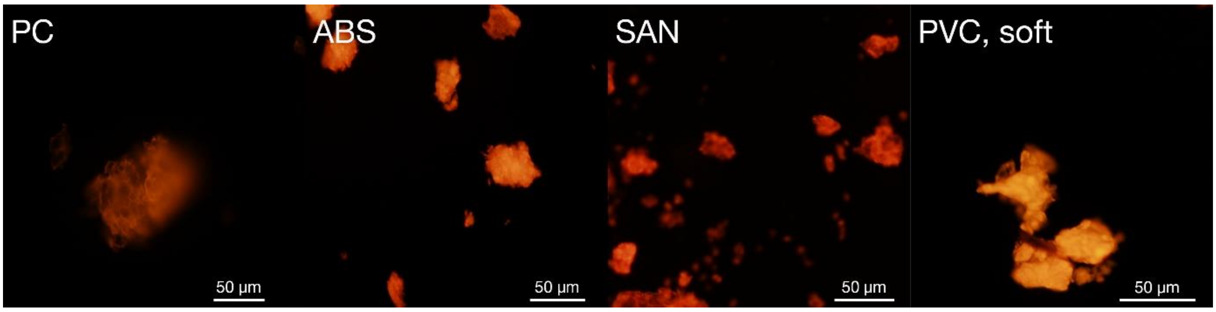

Fig. S2. Fluorescence microscopy images of microplastic particles from plastic mix B: Polycarbonate (PC), acrylonitrile-butadiene-styrene (ABS), styrene-acrylonitrile copolymer (SAN) and soft polyvinylchloride (PVC, soft). The particles were not stained with fluorescence dye, but images show their auto-fluorescence in order to increase the contrast. Particles were imaged under an Olympus BX61 with GFP-LP filter (PC, ABS, SAN) or Texas Red filter (PVC, soft).

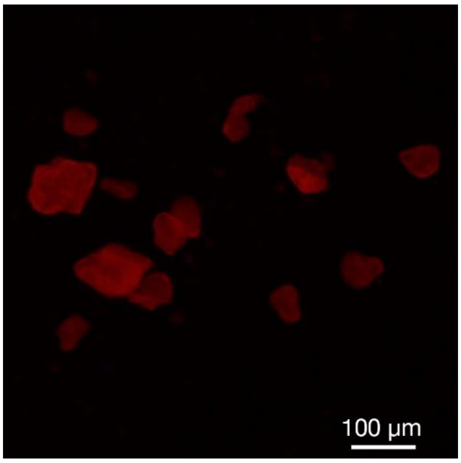

Fig. S3. Fluorescence microscopy images of microplastic particles from red fluorescent polymethyl methacrylate (PMMA). The particles were not stained with fluorescence dye, but images show their auto-fluorescence in order to increase the contrast. Particles were imaged under a Zeiss Axio Imager Z2m with Texas Red filter.

Table S1. Overview over the physical properties of the used polymers as well as the resulting particle sizes after the last grinding step. Size is given ± standard deviation.

polymer	plastic mix	manufacturers designation	density [g/cm3]	particle size [µm]	manufacturer
Polycarbonate with phosgene or diphenyl carbonate (PC)	A	Panlite L 1250-Y	1.20	28.20 ± 18.28	Teijin Kasei America, Inc.
Polyvinyl chloride (PVC), rigid	A	Troilit® VB 537-HE	1.43	27.54 ± 19.86	Granulat GmbH, Germany
Polyethylene terephthalate (PET)	A	- **	1.37−1.45*	39.18 ± 37.31	- **
Polyamide family (PA	A	Ultramid® B27 E	1.12-1.15	72.55 ± 74.68	BASF SE, Germany
Acrylonitrile-butadiene-styrene (ABS) terpolymer	B	Novodur P2MD	1.030	31.40 ± 21.23	Styrolution Group GmbH, Germany
Polyoxymethylene (POM) homopolymer	B	Delrin 500	1.41-1.61*	27.86 ± 22.65	DuPont
Polyvinyl chloride (PVC), plasticized	B	TRIOLIT® VB 637-WE/1	1.183	48.51 ± 55.53	Granulat GmbH, Germany
Styrene Acrylonitrile (SAN)	B	Luran 368R	1.080	22.32 ± 10.81	Styrolution Group GmbH, Germany
Red fluorescent, polymethyl methacrylate (PMMA)	preliminary experiment only			29.50 ± 26.00	unknown

* Density was not available in the specification sheets of the manufacturer, therefore values are taken from Hidalgo-Ruz et al. (2012).

** Polyethylen terephthalat particles were generated out of several beverage bottles

Preparation of the plastic particle stock suspensions

The stock suspension of the two plastic treatments as well as the fluorescent microplastic particles were prepared by the addition of 10 mg of powder from each plastic type to 20 ml filtered semi-artificial medium (Filter: GPWP, 0.22 µm, Merck Millipore, Germany) in a 50 ml Falcon Tube (VWR International GmbH, Germany). The suspension was vortexed multiple times, rinsed into a 500 ml glass bottle, filled with filtered semi-artificial medium to 500 ml and mixed for 48h.

Particle quantification

Quantification of algae particle concentration

In order to supply microplastic particles in a defined algae particle to microplastic particle ratio, the amount of algae particles in a standardized amount of food (1 mg C) was quantified using a Cell Counter & Analyzer System 1 (Schärfe System GmbH, Germany).

After harvesting algae food suspension is stored in a refrigerator at 10°C to sediment. *Daphnia* are then fed with an algae suspension of an age of 24h and 48h after harvesting. As *S. obliquus* builds cell packages of up to 8 cells which disaggregate with time and the Cell Counter & Analyzer System 1 cannot distinguish between single cells and cell aggregates, the food suspension was measured in algae food suspension of different time points after harvesting (fresh, 24h, 48h). From each of these three time points five samples were measured with three technical replicates and averaged. The approximate algae particle count over all time points was 30.000.000 algae particles for 1 mg C, for detailed values please refer to Table S2

Table S2. Algae particle counts and total volume of *S. obliquus* for 1mg C/L as average ± standard deviation.

	total volume [ml]	particle count/ml	average particle count/ml
**Fresh**	$5.5 \times 10^6 \pm 2.2 \times 10^5$	$17.7 \times 10^6 \pm 3.1 \times 10^5$	
**24h**	$4.7 \times 10^6 \pm 2.6 \times 10^5$	$37.1 \times 10^6 \pm 1.6 \times 10^5$	$28.95 \times 10^6 \pm 8.6 \times 10^6$
**48h**	$4.2 \times 10^6 \pm 5.4 \times 10^5$	$32.1 \times 10^6 \pm 2.0 \times 10^6$	

Particle quantification concentration of the plastic stock suspensions

For the quantification of the particle concentration of plastic mix A and B stock suspension it was necessary to stain the microplastic particles of plastic mix A and B prior the quantification with fluorescence microscopy. Otherwise it is not possible to distinguish them from other particles (dust, air bubbles, etc.). This was solely performed with a small subsample of the stock suspension of plastic mix A and B, but not in the feeding experiment itself. Additionally this staining step was skipped for the red fluorescent particles as these were inherently fluorescent.

Nile red staining of the plastic particles in the stock suspension of plastic mix A and B for the particle concentration quantification

A subsample of 1.5 ml of the stock suspension of plastic mix A and B were transferred to a 2 ml Eppendorf tube (Eppendorf AG, Germany), then 0.5 ml filtered isopropanol (60%) was added. All polymers in plastic mix A and B are stable against isopropanol (Bürkle GmbH 2011). The mixture was centrifuged for 10 min at 13.3 rpm and the supernatant removed with a glass pipette. The pellets were dyed with 300 µl nile red for 30 min and centrifuged for 1 min at 13.3 rpm, washed two times with isopropanol (60%) and centrifuged at 13.3 rpm and subsequently washed two times with H_2O (bidest) and centrifuged for 4 min at 13.3 rpm.

Quantification of plastic particle concentration in the stock suspension

18 µL of each stock suspension were filled in a Fuchs-Rosenthal counting chamber. Using a Zeiss Axioplan (40x objective, Carl Zeiss GmbH, Germany) and the analyzing software

AxioVision (Carl Zeiss GmbH, Germany) five images were generated from each of the stock suspensions and the fluorescent particles.

Supplied volumes

The necessary volumes of the stock suspensions of plastic mix A and plastic mix B to supply plastic particles in a concentration which corresponds to 1% of the algae particles of a standardized feeding of 1 mg C/L were calculated and are given in Table S4.

The two groups exposed to red fluorescent microplastic particles were supplied with 0.017 ml and 0.170 ml of the red fluorescent particles stock suspension. The concentration of the stock suspension was 6.703.098 particles/ml (Table S3) resulting in a particle concentration of 5697.6 particles/ml and 569.76 particles/ml which corresponds to 10% and 1% of the algae particles of a standardized feeding of 1 mg C/L.

Table S3. Quantification results of the amount of microplastic particles in the stock suspension of plastic mix A, the plastic mix B and the red fluorescent microplastic particles

**Stock suspension**	**Particles per ml**
plastic mix A	357 850
plastic mix B	237 813
red fluorescent plastic	6 703 098

Table S4. Volumes of the stock suspensions of plastic mix A and B supplied into the experimental units for a standardized feeding of 1 mg C/L.

**Medium [ml]**	**algae particle in 1 mg C**	**particle number that corresponds to 1% of algae particles of 1mg C**	**necessary volume for a particle concentration that corresponds to 1% of algae particles of 1mg C**	
			**plastic mix A [ml]**	**plastic mix B [ml]**
**1,000**	28 946 667			
**1,800**	52 104 000	521 040	1.456	2.191
**200**	5 789 350	57893.5	0.162	0.2435

References

Bürkle GmbH. 2011. Chemische Beständigkeit von Kunststoffen. Bad Bellingen, Germany.

Hidalgo-Ruz, V., Gutow, L., Thompson, R. C., Thiel, M. 2012. Microplastics in the marine environment: A review of the methods used for identification and quantification. Environmental Science & Technology 6(46): 3060–3075.
