# Supplementary material for: Do microplastic particles affect Daphnia magna at the morphological, life history and molecular level?
Source: PLoS One. 2017 Nov 16;12(11):e0187590. doi: 10.1371/journal.pone.0187590 (PMC5690657; doi:10.1371/journal.pone.0187590)
Supplement: S2 File — (PDF) [file pone.0187590.s005.pdf]

S2 File. Preliminary experiment: Ingestion of microplastic particles and establishment of experimental concentration

Materials and methods

The preliminary ingestion experiment was conducted with the *D. magna* clone Max4 which was exposed to red fluorescent polymethyl methacrylate microplastic particles (29.5 ± 26 µm, PMMA). Similar to the particles of both plastic mixes, used in the latter experiment, these particles are non-buoyant and sink to the bottom of the glasses and had a comparable irregular shape. *Daphnia* were exposed to two concentrations of red fluorescent microplastic particles in relation to algae particles. These corresponded to an amount of plastic particles of 1% and 10% of the algae particles.

Animals of the same age (carrying the second clutch) were kept individually in 250 ml glass jars with 200 ml semi-artificial medium based on ultrapure water, phosphate buffer and trace elements (Rabus & Laforsch 2011), each treatment (control, 1% and 10% microplastic particles) contained ten replicates. All experimental glasses were provided with 2 mg C/L every other day. The two groups exposed to red fluorescent microplastic particles were additionally supplied with red fluorescent particles resulting in a particle concentration of 569.8 particles/ml (1%) and 5697.6 (10%) particles/ml, respectively. Semi-artificial medium, food and the microplastic particles for both treatments were placed in the glass jars. After 24h the experimental animals were introduced, in order to allow algae and plastic particles to sink to the ground of the experimental glasses. Water exchange was performed every other day. After four days, the animals were individually anesthetized with CO_2, fixed in 8% formaldehyde with 6.8% sucrose over 48 hours, placed on microscopy slides with vectashield antifade mounting medium (Vector Laboratories, USA) and imaged under a fluorescence microscope Olympus BX61 (Olympus GmbH, Germany) using fluorescence excitation with GFP-LP and Texas Red filters. The red fluorescent particles were counted. For a comparison of the realistic

concentration with 1% plastic in the food we fed one individual with an unrealistically high microplastic to algae particle ratio of approximately 1:1. This animal is shown in Fig S2.

Results

The ingestion experiments using red fluorescent microplastic particles provided evidence for an uptake of microplastic particles into the digestive tract of *D. magna* after an exposure to red fluorescent microplastic particles in a concentration which corresponds to 10% and 1% of the provided algae particles (Fig. S1). At both concentration levels 100% of the individuals fed with red fluorescent particles had microplastic particles in the gut system. In contrast, no microplastic particles were detected in the gut system of the control animals. Animals fed with a plastic concentration which corresponds to 1% of the algae particles had an amount of 33 ± 22 particles (mean ± SD, min:6, max:68) in the digestive tract at the end of the experiment (Fig. S1B). In contrast, feeing with a plastic concentration which corresponds to 10% of the algae particles resulted in 190 ± 107 particles (mean ± SD, min:96, max:399) in the digestive tract (Fig. S1C). Comparing the rather realistic concentration with 1% plastic in the food with one individual fed with an unrealistically high microplastic to algae particle ratio of approximately 1:1, the digestive tract was filled with red fluorescent particles and it was not possible to distinguish between single microplastic particles (Fig. S1D).

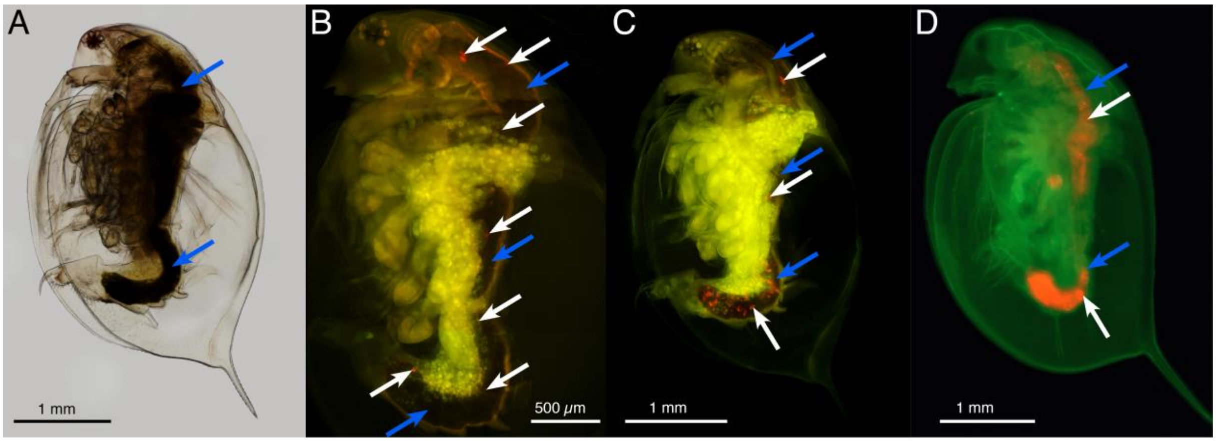

Fig. S1. Ingestion of red fluorescent microplastic particles by *D. magna*.
(A) Brightfield image of *D.* magna, with a digestive tract filled with food (algae particle). (B*)* *D. magna* which ingested red fluorescent microplastic particles in a concentration which corresponds to 1% of the supplied algae particles. (C) *D. magna* which was fed in a concentration which corresponds to 10% of the supplied algae particles. (D) *D. magna* fed with an approximate algae particle to microplastic particle ratio of 1:1. The digestive tract was filled with fluorescent microplastic particles. Single particles are hard to differentiate. The fluorescent images were produced at different focal planes and using different fluorescence excitations (GFP-LP, Texas Red) and then merged. The digestive tract is highlighted with a blue arrow, whereas red fluorescent particles are marked with white arrows.

References:

Rabus, M. & Laforsch, C. 2011. Growing large and bulky in the presence of the enemy: *Daphnia magna* gradually switches the mode of inducible morphological defences. Functional Ecology 25(5): 1137-1143.
