# Supplementary material for: Do microplastic particles affect Daphnia magna at the morphological, life history and molecular level?
Source: PLoS One. 2017 Nov 16;12(11):e0187590. doi: 10.1371/journal.pone.0187590 (PMC5690657; doi:10.1371/journal.pone.0187590)
Supplement: S3 File — (PDF) [file pone.0187590.s006.pdf]

S3 File. Supporting results: Evaluation of the gene expression after 48h of exposure to microplastic particles

Reference gene evaluation using geNorm analysis, M and CV values

Table S1. GeNorm M value (M) and their coefficient of variation on the normalized relative quantities (CV) of the used reference genes.

Clone	Gene	M	CV
**Bl2.2**	SDH	0.281	0.117
	TBP	0.204	0.139
	UBC	0.299	0.143
**K34J**	GAPDH	0.321	0.103
	UBC	0.318	0.102
	-		
**Max4**	GAPDH	0.318	0.112
	STX16	0.369	0.193
	UBC	0.319	0.142

Differential gene expression of the clones BL2.2, K34J and Max4

Table S2. Relative gene expression differences of the clone BL2.2 after normalization to the reference genes SDH, TBP and UBC.

Target	Treatment	Relative gene expression (mean)	95% value ci low	95% value ci high
Flot	control	1	0.826	1
	plastic mix A	0.886	0.819	0.886
	plastic mix B	0.982	0.762	0.982
GST	control	1	0.952	1
	plastic mix A	1.023	0.924	1.023
	plastic mix B	0.897	0.844	0.897
HSP60	control	1	0.864	1
	plastic mix A	1.295	1.106	1.295
	plastic mix B	1.156	1.054	1.156
HSP70	control	1	0.957	1
	plastic mix A	0.675	0.508	0.675
	plastic mix B	0.888	0.816	0.888
JHE	control	1	0.764	1
	plastic mix A	0.919	0.736	0.919
	plastic mix B	1.264	0.88	1.264
MetA	control	1	0.837	1
	plastic mix A	1.15	0.819	1.15
	plastic mix B	0.899	0.673	0.899
MetB	control	1	0.754	1
	plastic mix A	0.918	0.631	0.918
	plastic mix B	0.7	0.586	0.7
Act	control	1	0.854	1
	plastic mix A	0.717	0.623	0.717
	plastic mix B	0.782	0.692	0.782
aTub	control	1	0.895	1
	plastic mix A	0.605	0.473	0.605
	plastic mix B	0.552	0.504	0.552
GAPDH	control	1	0.901	1
	plastic mix A	0.839	0.724	0.839
	plastic mix B	0.709	0.604	0.709
SERCA	control	1	0.699	1
	plastic mix A	0.446	0.257	0.446
	plastic mix B	0.576	0.445	0.576
STX16	control	1	0.826	1
	plastic mix A	0.845	0.655	0.845
	plastic mix B	0.643	0.523	0.643

Table S3. Results of univariate ANOVA corrected for multiple testing by false discovery rate multiple comparison method (Benjamini & Hochberg 1995) and Tukey-Kramer to correct for pairwise group comparisons with qbase+ 2.6 for the clone BL2.2.

	ANOVA		Pairwise comparisons		
Target	p	r^2	Treatment A	Treatment B	Significant
Flot	0.47	0.096	control	plastic mix A	No
			control	plastic mix B	No
			plastic mix A	plastic mix B	No
GST	**0.02**	0.444	control	plastic mix A	No
			control	plastic mix B	**Yes**
			plastic mix A	plastic mix B	**Yes**
HSP60	**0.02**	0.447	control	plastic mix A	**Yes**
			control	plastic mix B	No
			plastic mix A	plastic mix B	No
HSP70	**0.01**	0.542	control	plastic mix A	**Yes**
			control	plastic mix B	No
			plastic mix A	plastic mix B	**Yes**
JHE	0.20	0.221	control	plastic mix A	No
			control	plastic mix B	No
			plastic mix A	plastic mix B	No
MetA	0.35	0.149	control	plastic mix A	No
			control	plastic mix B	No
			plastic mix A	plastic mix B	No
MetB	0.14	0.266	control	plastic mix A	No
			control	plastic mix B	No
			plastic mix A	plastic mix B	No
Act	**0.01**	0.568	control	plastic mix A	**Yes**
			control	plastic mix B	**Yes**
			plastic mix A	plastic mix B	No
aTub	**0.00**	0.768	control	plastic mix A	**Yes**
			control	plastic mix B	**Yes**
			plastic mix A	plastic mix B	No
GAPDH	**0.01**	0.574	control	plastic mix A	No
			control	plastic mix B	**Yes**
			plastic mix A	plastic mix B	No
SERCA	**0.02**	0.475	control	plastic mix A	**Yes**
			control	plastic mix B	No
			plastic mix A	plastic mix B	No
STX16	**0.02**	0.476	control	plastic mix A	No
			control	plastic mix B	**Yes**
			plastic mix A	plastic mix B	No

Table S4. Relative gene expression differences of the clone K34J after normalization to the reference genes GAPDH and UBC.

Target	Treatment	Relative gene expression (mean)	95% value ci low	95% value ci high
Flot	control	1	0.794	1.259
	plastic mix A	0.899	0.64	1.263
	plastic mix B	0.912	0.649	1.28
GST	control	1	0.896	1.116
	plastic mix A	1.08	0.97	1.202
	plastic mix B	0.85	0.677	1.067
HSP60	control	1	0.874	1.145
	plastic mix A	1.007	0.663	1.527
	plastic mix B	1.154	1.086	1.227
HSP70	control	1	0.704	1.42
	plastic mix A	0.809	0.566	1.156
	plastic mix B	0.821	0.428	1.574
JHE	control	1	0.661	1.514
	plastic mix A	0.903	0.612	1.332
	plastic mix B	0.755	0.412	1.385
MetA	control	1	0.768	1.302
	plastic mix A	1.18	0.885	1.572
	plastic mix B	1.276	0.982	1.658
MetB	control	1	0.794	1.259
	plastic mix A	1.063	0.878	1.287
	plastic mix B	1.036	0.738	1.454
Act	control	1	0.833	1.2
	plastic mix A	0.833	0.654	1.06
	plastic mix B	0.833	0.577	1.202
aTub	control	1	0.784	1.276
	plastic mix A	0.855	0.616	1.187
	plastic mix B	0.836	0.46	1.519
SERCA	control	1	0.758	1.32
	plastic mix A	0.806	0.59	1.101
	plastic mix B	0.586	0.191	1.797
SDH	control	1	0.877	1.141
	plastic mix A	1.006	0.801	1.265
	plastic mix B	0.917	0.722	1.166
STX16	control	1	0.934	1.071
	plastic mix A	1.031	0.86	1.237
	plastic mix B	1.003	0.756	1.329
TBP	control	1	0.855	1.17
	plastic mix A	0.979	0.759	1.263
	plastic mix B	1.109	0.838	1.468

Table S5. Results of univariate ANOVA corrected for multiple testing by false discovery rate multiple comparison method (Benjamini & Hochberg 1995) and Tukey-Kramer to correct for pairwise group comparisons with qbase+ 2.6 for the clone K34J.

	Main test		Pairwise comparisons		
Target	p	r^2	Treatment A	Treatment B	Significant
Flot	0.94	0.03	control	plastic mix A	No
			control	plastic mix B	No
			plastic mix A	plastic mix B	No
GST	0.56	0.343	control	plastic mix A	No
			control	plastic mix B	No
			plastic mix A	plastic mix B	Yes
HSP60	0.91	0.081	control	plastic mix A	No
			control	plastic mix B	No
			plastic mix A	plastic mix B	No
HSP70	0.91	0.052	control	plastic mix A	No
			control	plastic mix B	No
			plastic mix A	plastic mix B	No
JHE	0.91	0.072	control	plastic mix A	No
			control	plastic mix B	No
			plastic mix A	plastic mix B	No
MetA	0.91	0.156	control	plastic mix A	No
			control	plastic mix B	No
			plastic mix A	plastic mix B	No
MetB	0.96	0.012	control	plastic mix A	No
			control	plastic mix B	No
			plastic mix A	plastic mix B	No
Act	0.91	0.115	control	plastic mix A	No
			control	plastic mix B	No
			plastic mix A	plastic mix B	No
aTub	0.91	0.046	control	plastic mix A	No
			control	plastic mix B	No
			plastic mix A	plastic mix B	No
SERCA	0.91	0.118	control	plastic mix A	No
			control	plastic mix B	No
			plastic mix A	plastic mix B	No
SDH	0.91	0.053	control	plastic mix A	No
			control	plastic mix B	No
			plastic mix A	plastic mix B	No
STX16	0.96	0.006	control	plastic mix A	No
			control	plastic mix B	No
			plastic mix A	plastic mix B	No
TBP	0.91	0.066	control	plastic mix A	No
			control	plastic mix B	No
			plastic mix A	plastic mix B	No

Table S6. Relative gene expression differences of the clone Max4 after normalization to the reference genes GAPDH, STX16 and UBC.

Target	Treatment	Relative gene expression (mean)	95% value ci low	95% value ci high
Flot	control	1	0.69	1.448
	plastic mix A	1.503	1.109	2.037
	plastic mix B	1.237	1.08	1.416
GST	control	1	0.904	1.106
	plastic mix A	1.143	0.952	1.373
	plastic mix B	1.168	1.051	1.299
HSP60	control	1	0.848	1.179
	plastic mix A	1.233	1.032	1.474
	plastic mix B	1.361	1.238	1.497
HSP70	control	1	0.752	1.33
	plastic mix A	1.15	0.857	1.542
	plastic mix B	1.067	0.841	1.354
JHE	control	1	0.678	1.475
	plastic mix A	1.182	0.674	2.071
	plastic mix B	1.264	1.038	1.539
MetA	control	1	0.814	1.229
	plastic mix A	0.893	0.669	1.191
	plastic mix B	0.86	0.683	1.084
MetB	control	1	0.87	1.149
	plastic mix A	1.272	0.874	1.85
	plastic mix B	1.278	1.186	1.378
Act	control	1	0.747	1.339
	plastic mix A	1.458	1.1	1.931
	plastic mix B	1.357	1.129	1.632
aTub	control	1	0.625	1.601
	plastic mix A	0.969	0.692	1.355
	plastic mix B	0.834	0.584	1.192
SDH	control	1	0.793	1.261
	plastic mix A	1.591	1.252	2.021
	plastic mix B	1.589	1.318	1.916
SERCA	control	1	0.639	1.564
	plastic mix A	2.543	1.537	4.207
	plastic mix B	1.669	1.122	2.482
TBP	control	1	0.817	1.224
	plastic mix A	1.167	0.944	1.443
	plastic mix B	1.174	0.963	1.43

Table S7. Results of univariate ANOVA corrected for multiple testing by false discovery rate multiple comparison method (Benjamini & Hochberg 1995) and Tukey-Kramer to correct for pairwise group comparisons with qbase+ 2.6 for the clone Max4.

	Main test		Pairwise comparisons		
Target	p	r^2	Treatment A	Treatment B	Significant
Flot	0.17	0.307	control	plastic mix A	No
			control	plastic mix B	No
			plastic mix A	plastic mix B	No
GST	0.24	0.255	control	plastic mix A	No
			control	plastic mix B	No
			plastic mix A	plastic mix B	No
HSP60	**0.03**	0.492	control	plastic mix A	No
			control	plastic mix B	**Yes**
			plastic mix A	plastic mix B	No
HSP70	0.73	0.054	control	plastic mix A	No
			control	plastic mix B	No
			plastic mix A	plastic mix B	No
JHE	0.73	0.071	control	plastic mix A	No
			control	plastic mix B	No
			plastic mix A	plastic mix B	No
MetA	0.73	0.083	control	plastic mix A	No
			control	plastic mix B	No
			plastic mix A	plastic mix B	No
MetB	0.24	0.239	control	plastic mix A	No
			control	plastic mix B	No
			plastic mix A	plastic mix B	No
Act	0.13	0.348	control	plastic mix A	Yes
			control	plastic mix B	No
			plastic mix A	plastic mix B	No
aTub	0.73	0.051	control	plastic mix A	No
			control	plastic mix B	No
			plastic mix A	plastic mix B	No
SDH	**0.03**	0.565	control	plastic mix A	**Yes**
			control	plastic mix B	**Yes**
			plastic mix A	plastic mix B	No
SERCA	**0.03**	0.486	control	plastic mix A	**Yes**
			control	plastic mix B	No
			plastic mix A	plastic mix B	No
TBP	0.49	0.148	control	plastic mix A	No
			control	plastic mix B	No
			plastic mix A	plastic mix B	No

References:

Benjamini, Y. & Hochberg, Y. 1995. Controlling the false discovery rate: A practical and powerful approach to multiple testing. Journal of the Royal Statistical Society. Series B (Methodological) 57(1): 289-300.
