# Supplementary material for: Do microplastic particles affect Daphnia magna at the morphological, life history and molecular level?
Source: PLoS One. 2017 Nov 16;12(11):e0187590. doi: 10.1371/journal.pone.0187590 (PMC5690657; doi:10.1371/journal.pone.0187590)
Supplement: S4 File — (PDF) [file pone.0187590.s007.pdf]

S4 File. Supporting results: Morphology and life-history parameters

Morphology parameters of adult Daphnia magna

Body length

Table S1. PERMANOVA test results of the body length of the three *D. magna* clones BL2.2, K34J and Max4. PERMANOVA with the time point of the measurements (48h after primiparity and the carrying time of the 3rd and 5th clutch) as additional fixed factor.

	PERMANOVA					
fixed factor	Body length (response variable)			time point (additional fixed factor)		
	Pseudo-F	df	p(MC)	Pseudo-F	df	p(MC)
BL2.2	0.60959	6	0.719	1177.1	2	<0.001
K34J	0.70056	6	0.649	1914.9	2	<0.001
Max4	1.4875	6	0.186	1189.6	2	<0.001

Table S2. PERMDISP test results of the body length of the three *D. magna* clones BL2.2, K34J and Max4. Individual PERMDISP tests were performed for each time point of the measurements (48h after primiparity and the carrying time of the 3rd and 5th clutch).

		PERMDISP						
		Body length (response variable)				Pairwise comparisons		
	time point	F	df_1	df_2	p	Treatment A	Treatment B	p
BL2.2	48h after primiparity	0.2125	2	51	0.832	control	plastic mix A	-
							plastic mix B	-
	3rd clutch	2.9507	2	51	0.064	control	plastic mix A	-
							plastic mix B	-
	5th clutch	0.4148	2	51	0.722	control	plastic mix A	-
							plastic mix B	-
K34J	48h after primiparity	2.7359	2	51	0.077	control	plastic mix A	-
							plastic mix B	-
	3rd clutch	1.4402	2	50	0.253	control	plastic mix A	-
							plastic mix B	-
	5th clutch	1.4714	2	50	0.279	control	plastic mix A	-
							plastic mix B	-
Max4	48h after primiparity	12.166	2	51	**<0.001**	control	plastic mix A	**0.005**
							plastic mix B	**0.001**
	3rd clutch	1.5714	2	50	0.224	control	plastic mix A	-
							plastic mix B	-
	5th clutch	2.6134	2	48	0.089	control	plastic mix A	-
							plastic mix B	-

Body width

Table S3. PERMANOVA test results of the body width of the three *D. magna* clones BL2.2, K34J and Max4. PERMANOVA was performed with the time point of the measurements (48h after primiparity and the carrying time of the 3rd and 5th clutch) as additional fixed factor and the body length as covariate to compensate for size-dependent differences. The interaction term body width × body length was non-significant for all clones.

	PERMANOVA								
Fixed factor	Body width (response variable)			Body length (covariate)			Time point (additional fixed factor)		
	Pseudo-F	df	p(MC)	Pseudo-F	df	p(MC)	Pseudo-F	df	p(MC)
BL2.2	3.4489	6	**0.003**	8484.8	1	<0.001	9.1167	2	<0.001
K34J	1.237	6	0.304	10267	1	<0.001	57.215	2	<0.001
Max4	0.46547	6	0.831	6781.7	1	<0.001	12.674	2	<0.001

Table S4. Pairwise comparisons of the body width 48h after primiparity and the carrying time of the 3rd and 5th clutch.

		Pairwise comparisons		
	Time point	Treatment A	Treatment B	p(MC)
BL2.2	48h after primiparity	control	plastic mix A	0.752
			plastic mix B	0.580
	3rd clutch	control	plastic mix A	*0.072*
			plastic mix B	**0.023**
	5th clutch	control	plastic mix A	0.182
			plastic mix B	0.134

Table S5. PERMDISP test results of the body width of the three *D. magna* clones BL2.2, K34J and Max4. Individual PERMDISP tests were performed for each time point of the measurements (48h after primiparity and the carrying time of the 3rd and 5th clutch).

		PERMDISP						
		Body width (response variable)				Pairwise comparisons		
	Time point	F	df_1	df_2	p	Treatment A	Treatment B	p
BL2.2	48h after primiparity	0.62082	2	51	0.570	control	plastic mix A	-
							plastic mix B	-
	3rd clutch	0.19698	2	51	0.832	control	plastic mix A	-
							plastic mix B	-
	5th clutch	0.36649	2	51	0.715	control	plastic mix A	-
							plastic mix B	-
K34J	48h after primiparity	0.45502	2	51	0.640	control	plastic mix A	-
							plastic mix B	-
	3rd clutch	0.40192	2	50	0.677	control	plastic mix A	-
							plastic mix B	-
	5th clutch	0.14675	2	50	0.892	control	plastic mix A	-
							plastic mix B	-
Max4	48h after primiparity	0.22638	2	51	0.812	control	plastic mix A	-
							plastic mix B	-
	3rd clutch	0.09724	2	50	0.917	control	plastic mix A	-
							plastic mix B	-
	5th clutch	0.36032	2	48	0.692	control	plastic mix A	-
							plastic mix B	-

Tail spine length

Table S6. PERMANOVA test results of the tail spine length of the three *D. magna* clones BL2.2, K34J and Max4. PERMANOVA was performed with the time point of the measurements (48h after primiparity and the carrying time of the 3rd and 5th clutch) as additional fixed factor and the body length as covariate to compensate for size-dependent differences. The interaction term body width × body length was non-significant for all clones.

	PERMANOVA								
fixed factor	Tail spine length (response variable)			Body length (covariate)			Tine point (additional fixed factor)		
	Pseudo-F	df	p(MC)	Pseudo-F	df	p(MC)	Pseudo-F	df	p(MC)
BL2.2	3.2958	6	**0.005**	9.9969	1	0.002	53.9430	2	<0.001
K34J	0.7002	6	0.654	4.7712	1	0.031	7.1971	2	<0.001
Max4	2.6069	6	**0.020**	13.0300	1	<0.001	4.3518	2	0.014

Table S7. Pairwise comparisons of the tail spine length 48h after primiparity and the carrying time of the 3rd and 5th clutch. For Max4 the interaction term was significant, therefore, analysis was additionally performed without the body length as covariate.

		Pairwise comparisons		
		Treatment A	Treatment B	p(MC)
BL2.2	48h after primiparity	control	plastic mix A	0.948
			plastic mix B	0.776
	3rd clutch	control	plastic mix A	0.347
			plastic mix B	0.622
	5th clutch	control	plastic mix A	**<0.001**
			plastic mix B	0.743
Max4	48h after primiparity	control	plastic mix A	**0.012**
			plastic mix B	0.199
	3rd clutch	control	plastic mix A	0.301
			plastic mix B	0.657
	5th clutch	control	plastic mix A	0.143
			plastic mix B	0.680

Table S8. PERMDISP test results of the tail spine length of the three *D. magna* clones BL2.2, K34J and Max4. Individual PERMDISP tests were performed for each time point of the measurements (48h after primiparity and the carrying time of the 3rd and 5th clutch).

		PERMDISP						
		Tail spine length (response variable)				Pairwise comparisons		
	Time point	F	df_1	df_2	p	Treatment A	Treatment B	p
BL2.2	48h after primiparity	2.5603	2	48	0.089	control	plastic mix A	-
							plastic mix B	-
	3rd clutch	1.2855	2	49	0.325	control	plastic mix A	-
							plastic mix B	-
	5th clutch	1.9561	2	51	0.210	control	plastic mix A	-
							plastic mix B	-
K34J	48h after primiparity	1.2777	2	61	0.489	control	plastic mix A	-
							plastic mix B	-
	3rd clutch	0.0381	2	50	0.969	control	plastic mix A	-
							plastic mix B	-
	5th clutch	0.98507	2	49	0.543	control	plastic mix A	-
							plastic mix B	-
Max4	48h after primiparity	1.4647	2	50	0.340	control	plastic mix A	-
							plastic mix B	-
	3rd clutch	1.2315	2	48	0.307	control	plastic mix A	-
							plastic mix B	-
	5th clutch	3.5844	2	45	**0.037**	control	plastic mix A	0.208
							plastic mix B	**0.012**

Reproduction of Daphnia magna

Number of neonates

Table S9. PERMANOVA test results of the produced number of neonates during the experimental period of the three *D. magna* clones BL2.2, K34J and Max4. PERMANOVA with the time point of the measurements (1st clutch – 5th clutch) as additional fixed factor.

	PERMANOVA					
fixed factor	Number of neonates (response variable)			Time point (additional fixed factor)		
	Pseudo-F	df	p(MC)	Pseudo-F	df	p(MC)
BL2.2	1.329	6	0.212	263.14	4	<0.001
K34J	0.84693	6	0.581	300.28	4	<0.001
Max4	0.33732	6	0.969	89.631	4	<0.001

Table S10. PERMDISP test results of the produced number of neonates of the three *D. magna* clones BL2.2, K34J and Max4. Individual PERMDISP tests were performed for each time point of the measurements (1st clutch – 5th clutch).

		PERMDISP						
		Number of neonates (response variable)				Pairwise comparisons		
	clutch	F	df_1	df_2	p	Treatment A	Treatment B	p
BL2.2	1	0.7847	2	51	0.467	control	plastic mix A	-
							plastic mix B	-
	2	1.4774	2	51	0.278	control	plastic mix A	-
							plastic mix B	-
	3	1.0144	2	51	0.370	control	plastic mix A	-
							plastic mix B	-
	4	0.4670	2	51	0.638	control	plastic mix A	-
							plastic mix B	-
	5	2.0373	2	51	0.148	control	plastic mix A	-
							plastic mix B	-
K34J	1	0.7630	2	51	0.543	control	plastic mix A	-
							plastic mix B	-
	2	2.0298	2	50	0.142	control	plastic mix A	-
							plastic mix B	-
	3	1.6565	2	50	0.219	control	plastic mix A	-
							plastic mix B	-
	4	1.9104	2	50	0.164	control	plastic mix A	-
							plastic mix B	-
	5	0.3750	2	49	0.708	control	plastic mix A	-
							plastic mix B	-
Max4	1	0.2113	2	51	0.827	control	plastic mix A	-
							plastic mix B	-
	2	0.1231	2	51	0.884	control	plastic mix A	-
							plastic mix B	-
	3	0.2169	2	49	0.824	control	plastic mix A	-
							plastic mix B	-
	4	2.0160	2	48	0.162	control	plastic mix A	-
							plastic mix B	-
	5	0.9155	2	43	0.577	control	plastic mix A	-
							plastic mix B	-

Number of males

For the clone BL2.2 males occurred at the end of the experiment in the 5th clutch in two replicates of the control, in 3 replicates of the group fed with plastic mix A and in 4 replicates fed with plastic mix B. In the control 7.0 ± 7.2 % of the clutch were males. In the treatment fed with plastic mix A and B 2.9 ± 1.6 % and 6.8 ± 5.2 % of the clutch were males. In the clone Max4 also males started to appear in the 5th clutch. Herein 4 replicates in the control treatment with 55.4 ± 42.8 % males and 3 replicates of the treatment fed with plastic mix B with 69.7 ± 35.2 % males. In the clone K34J no males appeared.

Morphology parameters of the produced juveniles by Daphnia magna

Body length

Table S11. PERMANOVA test results of the body length of produced neonates of the three *D. magna* clones BL2.2, K34J and Max4 during the experimental period. Nested PERMANOVA was performed with the time point of the measurements (1st, 3rd & 5th clutch as additional fixed factor.

	Nested PERMANOVA					
fixed factor	Body length (response variable)			Tine point (additional fixed factor)		
	Pseudo-F	df	p(MC)	Pseudo-F	df	p(MC)
BL2.2	2.1107	6	*0.056*	397.81	2	**<0.001**
K34J	2.3641	6	**0.032**	397.21	2	**<0.001**
Max4	4.3105	6	**<0.001**	163.15	2	**<0.001**

Table S12. Pairwise comparisons of the body length of produced neonates in the 1st, 3rd and 5th clutch.

		Pairwise comparisons		
		Treatment A	Treatment B	p(MC)
BL2.2	1st clutch	control	plastic mix A	0.598
			plastic mix B	0.737
	3rd clutch	control	plastic mix A	*0.019*
			plastic mix B	0.269
	5th clutch	control	plastic mix A	0.480
			plastic mix B	*0.007*
K34J	1st clutch	control	plastic mix A	0.845
			plastic mix B	0.418
	3rd clutch	control	plastic mix A	**0.038**
			plastic mix B	*0.075*
	5th clutch	control	plastic mix A	0.971
			plastic mix B	**0.021**
Max4	1st clutch	control	plastic mix A	**0.012**
			plastic mix B	0.156
	3rd clutch	control	plastic mix A	0.377
			plastic mix B	**0.028**
	5th clutch	control	plastic mix A	0.665
			plastic mix B	*0.066*

Table S13. PERMDISP test results of the body length of produced neonates of the three *D. magna* clones BL2.2, K34J and Max4. Individual PERMDISP tests were performed for each time point of the measurements (1st, 3rd & 5th clutch).

		PERMDISP						
		Body length (response variable)				Pairwise comparisons		
	Time point	F	df_1	df_2	p	Treatment A	Treatment B	p
BL2.2	1st clutch	1.2324	2	267	0.359	control	plastic mix A	-
							plastic mix B	-
	3rd clutch	5.3292	2	267	**0.007**	control	plastic mix A	**0.003**
							plastic mix B	**0.025**
	5th clutch	2.7361	2	267	0.063	control	plastic mix A	-
							plastic mix B	-
K34J	1st clutch	1.945	2	267	0.141	control	plastic mix A	-
							plastic mix B	-
	3rd clutch	0.63512	2	262	0.557	control	plastic mix A	-
							plastic mix B	-
	5th clutch	0.43778	2	257	0.650	control	plastic mix A	-
							plastic mix B	-
Max4	1st clutch	3.0801	2	267	**0.045**	control	plastic mix A	0.077
							plastic mix B	**0.026**
	3rd clutch	4.6267	2	257	**0.017**	control	plastic mix A	**0.039**
							plastic mix B	0.577
	5th clutch	0.80095	2	225	0.506	control	plastic mix A	-
							plastic mix B	-

Body width

Table S14. PERMANOVA test results of the body width of produced neonates of the three *D. magna* clones BL2.2, K34J and Max4 during the experimental period. Nested PERMANOVA was performed with the time point of the measurements (1st, 3rd & 5th clutch as additional fixed factor and the body length as covariate to compensate for size-dependent differences. The interaction term body width × body length was non-significant for all clones.

	Nested PERMANOVA								
Fixed factor	Body width (response variable)			Body length (covariate)			Tine point (additional fixed factor)		
	Pseudo-F	df	p(MC)	Pseudo-F	df	p(MC)	Pseudo-F	df	p(MC)
BL2.2	3.0163	6	**0.007**	4683.2	1	**<0.001**	31.103	2	**<0.001**
K34J	1.5978	6	0.148	6059.8	1	**<0.001**	19.185	2	**<0.001**
Max4	2.1584	6	*0.050*	3443.4	1	**<0.001**	21.406	2	**0.014**

Table S15. Pairwise comparisons of body width of produced neonates in the 1st, 3rd and 5th clutch.

		Pairwise comparisons		
		Treatment A	Treatment B	p(MC)
BL2.2	1st clutch	control	plastic mix A	**0.001**
			plastic mix B	0.171
	3rd clutch	control	plastic mix A	0.150
			plastic mix B	0.160
	5th clutch	control	plastic mix A	0.178
			plastic mix B	0.996
Max	1st clutch	control	plastic mix A	0.724
			plastic mix B	*0.067*
	3rd clutch	control	plastic mix A	0.524
			plastic mix B	0.678
	5th clutch	control	plastic mix A	**0.011**
			plastic mix B	0.945

Table S16. PERMDISP test results of the body width of produced neonates of the three *D. magna* clones BL2.2, K34J and Max4. Individual PERMDISP tests were performed for each time point of the measurements (1st, 3rd & 5th clutch).

		PERMDISP						
		Body width (response variable)				Pairwise comparisons		
	time point	F	df_1	df_2	p(MC)	Treatment A	Treatment B	p
BL2.2	1st clutch	0.8576	2	267	0.440	control	plastic mix A	-
							plastic mix B	-
	3rd clutch	3.2170	2	267	**0.045**	control	plastic mix A	**0.037**
							plastic mix B	**0.032**
	5th clutch	3.6843	2	267	**0.029**	control	plastic mix A	**0.015**
							plastic mix B	0.562
K34J	1st clutch	1.0003	2	267	0.378	control	plastic mix A	-
							plastic mix B	-
	3rd clutch	2.8187	2	262	0.070	control	plastic mix A	-
							plastic mix B	-
	5th clutch	1.3571	2	257	0.260	control	plastic mix A	-
							plastic mix B	-
Max4	1st clutch	0.7907	2	267	0.461	control	plastic mix A	-
							plastic mix B	-
	3rd clutch	3.0048	2	257	0.057	control	plastic mix A	-
							plastic mix B	-
	5th clutch	1.0697	2	225	0.354	control	plastic mix A	-
							plastic mix B	-

Tail spine length

Table S17. PERMANOVA test results of the tail spine length of produced neonates of the three *D. magna* clones BL2.2, K34J and Max4 during the experimental period. Nested PERMANOVA was performed with the time point of the measurements (1st, 3rd & 5th clutch as additional fixed factor and the body length as covariate to compensate for size-dependent differences. The interaction term body width × body length was non-significant for all clones.

	nested PERMANOVA								
fixed factor	Tail spine length (response variable)			Body length (covariate)			Tine point (additional fixed factor)		
	Pseudo-F	df	p(MC)	Pseudo-F	df	p(MC)	Pseudo-F	df	p(MC)
BL2.2	0.67104	6	0.668	1993.9	1	<0.001	16.288	2	<0.001
K34J	0.48168	6	**0.043**	1298.5	1	<0.001	1.7538	2	0.170
Max4	3.0704	6	**0.008**	2193.2	1	<0.001	186.84	2	0.014

Table S18. Pairwise comparisons of the tail spine length of produced neonates in the 1st, 3rd and 5th clutch.

		Pairwise comparisons		
		Treatment A	Treatment B	p(MC)
K34J	1st clutch	control	plastic mix A	0.806
			plastic mix B	**0.012**
	3rd clutch	control	plastic mix A	0.453
			plastic mix B	**0.036**
	5th clutch	control	plastic mix A	1
			plastic mix B	0.336
Max4	1st clutch	control	plastic mix A	0.217
			plastic mix B	*0.057*
	3rd clutch	control	plastic mix A	**0.669**
			plastic mix B	0.411
	5th clutch	control	plastic mix A	**0.009**
			plastic mix B	0.461

Table S19. PERMDISP test results of the tail spine length of produced neonates of the three *D. magna* clones BL2.2, K34J and Max4. Individual PERMDISP tests were performed for each time point of the measurements (1st, 3rd & 5th clutch).

		PERMDISP						
		Tail spine length (response variable)				Pairwise comparisons		
	Time point	F	df_1	df_2	p	Treatment A	Treatment B	p
BL2.2	1st clutch	0.0412	2	267	0.963	control	plastic mix A	-
							plastic mix B	-
	3rd clutch	8.4265	2	267	**<0.001**	control	plastic mix A	**0.030**
							plastic mix B	**<0.001**
	5th clutch	1.6246	2	267	0.197	control	plastic mix A	-
							plastic mix B	-
K34J	1st clutch	1.6266	2	267	0.207	control	plastic mix A	-
							plastic mix B	-
	3rd clutch	0.4809	2	262	0.641	control	plastic mix A	-
							plastic mix B	-
	5th clutch	0.3706	2	257	0.716	control	plastic mix A	-
							plastic mix B	-
Max4	1st clutch	2.2055	2	267	0.118	control	plastic mix A	-
							plastic mix B	-
	3rd clutch	0.7788	2	257	0.464	control	plastic mix A	-
							plastic mix B	-
	5th clutch	1.3121	2	224	0.272	control	plastic mix A	-
							plastic mix B	-
